# A Comprehensive Review on Cellulose Nanofibers, Nanomaterials, and Composites: Manufacturing, Properties, and Applications

**DOI:** 10.3390/nano15050356

**Published:** 2025-02-25

**Authors:** Subin Antony Jose, Nicholas Cowan, Matthew Davidson, Giovanni Godina, Ian Smith, Justin Xin, Pradeep L. Menezes

**Affiliations:** Department of Mechanical Engineering, University of Nevada-Reno, Reno, NV 89557, USA; subinj@unr.edu (S.A.J.); niccowan651@gmail.com (N.C.); mtdavidson2004@gmail.com (M.D.); ggodina@unr.edu (G.G.); ipsmith@unr.edu (I.S.); justinx@unr.edu (J.X.)

**Keywords:** cellulose nanofibers, cellulose nanomaterials, cellulose-based composites, cellulose nanocrystals

## Abstract

Cellulose nanofibers (CNFs), cellulose nanomaterials (CNMs), and cellulose-based composites represent a convergence of material science, sustainability, and advanced engineering, paving the way for innovative and eco-friendly materials. This paper presents a comprehensive review of these materials, encompassing their extraction, preparation methods, properties, applications, and future directions. The manufacturing of CNFs and CNMs leverages diverse techniques—chemical, mechanical, and enzymatic—with each offering distinct advantages in tailoring material characteristics to meet specific needs. Strategies for functionalization and surface modification are detailed, highlighting their role in enhancing the properties of CNFs and composites while addressing challenges in scaling production to industrial levels. The structural, mechanical, thermal, optical, electrical, and biocompatibility properties of CNFs, CNMs, and their composites are explored, underscoring their versatility for applications across various industries. Cellulose-based composites, in particular, demonstrate exceptional tunable properties for specific uses, although achieving uniform dispersion remains a key technical hurdle. These materials have applications in packaging, automotive, aerospace, biomedical devices, energy storage, and environmental remediation. Emerging research trends emphasize the integration of CNFs and CNMs with advanced manufacturing technologies, promoting sustainable practices and life cycle considerations while advancing their commercialization potential. This rapidly evolving field holds immense promise for addressing global challenges by creating high-performance, and sustainable materials. This review is crucial in advancing the understanding of cellulose nanofibers, nanomaterials, and cellulose-based composites, providing valuable insights that will drive the development of sustainable, high-performance materials for a wide range of applications, ultimately addressing key global challenges.

## 1. Introduction

Cellulose, the most abundant natural polymer, is a crucial structural component of plant cell walls and a widely available renewable resource. Its biodegradability, high mechanical strength, and chemical stability make it an essential material in industries such as paper manufacturing, textiles, pharmaceuticals, food packaging, and biomedical applications [1]. Due to its sustainability and versatility, cellulose has been extensively researched for developing eco-friendly materials that can replace conventional synthetic polymers. Recent advancements have led to the emergence of nanocellulose, which includes cellulose nanofibers (CNFs), cellulose nanomaterials (CNMs), and cellulose nanocrystals (CNCs), offering superior mechanical, thermal, and barrier properties [2].

CNFs represent a groundbreaking class of nanomaterials derived from renewable cellulose sources, garnering significant attention for their remarkable properties and wide-ranging applications. CNFs typically have diameters ranging from 5 to 100 nm and lengths in the several micrometer range [3]. CNFs exhibit exceptional mechanical strength, biodegradability, and versatility, making them ideal for enhancing the performance of materials in lightweight, durable, and sustainable products [4,5]. These attributes have driven their adoption across diverse industries, including packaging, automotive, and biomedical sectors, highlighting their transformative potential and fueling efforts toward their exploration and commercialization [6].

CNMs encompass an array of cellulose-based nanostructures, including CNCs and other innovative forms of nanocellulose [4]. As one of the most abundant natural polymers on Earth, cellulose offers a sustainable and renewable resource base. CNCs generally have diameters of 3 to 20 nm and lengths of 100 to 500 nm [7]. The unique nanostructure of CNMs endows them with superior properties, such as high surface area, mechanical strength, and functional adaptability [6]. Their ability to be sourced from biomass aligns with global priorities for sustainable development, further solidifying their appeal in applications like nanocomposites, electronic devices, and environmental remediation [8].

Cellulose nanocomposites, in particular, have emerged as advanced materials that incorporate CNFs or CNCs into a matrix, typically a polymer, to significantly enhance mechanical, thermal, and barrier properties. CNFs act as effective reinforcement agents, improving the strength and stability of the matrix while maintaining a lightweight structure [4]. This synergy between cellulose nanostructures and the matrix material enables the development of composites with superior performance characteristics, including enhanced durability and resistance to environmental stresses. Achieving optimal dispersion of CNFs within the matrix is a key challenge, requiring careful design of the matrix material and fabrication processes to ensure uniform performance [9]. The adaptability of cellulose nanocomposites has made them suitable for applications spanning industries such as automotive, aerospace, medical devices, and environmental systems.

CNFs offer a sustainable and biodegradable alternative to synthetic nanomaterials and traditional materials for various applications. With their superior mechanical strength, CNFs are a competitive alternative to conventional reinforcement materials. However, their thermal stability is lower than that of carbon nanotubes (CNTs) and graphene [10]. Unlike non-biodegradable CNTs and polymer-based fibers, CNFs decompose naturally, reducing environmental impact. Their excellent gas barrier properties make them suitable for packaging and comparable to graphene but at a lower cost. While traditional polymers and glass fibers are easier to process, CNFs provide tunable surface chemistry for enhanced functionality [11].

This research paper provides a comprehensive exploration of cellulose nanofibers, nanomaterials, and nanocomposites. Although several review articles focus on the extraction methods of nanocellulose [12,13], this review offers a concise overview of the manufacturing processes of CNFs and CNMs, with a particular emphasis on extraction, preparation, and processing techniques aimed at optimizing their performance. The discussion extends to the structural, mechanical, and thermal properties of these materials, alongside their environmental impacts. The fabrication and reinforcement mechanisms of CNF-based nanocomposites are examined, addressing challenges such as achieving homogeneous dispersion and tailoring properties for specific applications. Finally, this paper highlights applications of CNFs and nanocomposites across various industries and considers future directions, emphasizing emerging trends, sustainability initiatives, and commercialization opportunities.

## 2. Manufacturing of Cellulose Nanofibers and Nanomaterials

The manufacturing of CNFs and CNMs relies on the utilization of organic, renewable materials. Cellulose, a natural biopolymer, is derived from various sustainable sources [4]. Common sources include wood, cotton, seeds, leaf fibers, and agricultural residues such as wheat and rice straw [6]. Among these, wood pulp and cotton are the most prevalent due to their high availability and substantial cellulose content, approximately 50% in wood and up to 90% in cotton [14,15]. In recent years, alternative cellulose sources, such as non-wood crops and aquatic plants, have gained attention for their advantages. These sources often feature shorter growth cycles and simplified manufacturing processes, making them attractive options for sustainable and cost-effective production. This diversification of cellulose feedstock expands the resource base and aligns with global efforts to promote environmentally friendly and economically viable materials.

### 2.1. Extraction and Preparation Methods

The preparation of CNFs and CNMs begins with the extraction of cellulose from its natural sources. A key step in this process is delignification, which involves the removal of lignin—a structural polymer found in the cell walls of plants—and other non-cellulosic materials through chemical treatment. This step purifies the cellulose fibers, preparing them for further processing. The CNFs consist of both amorphous and crystalline cellulose regions. Typically, they have a diameter ranging from 1 to 100 nm and a length between 500 and 2000 nm [16]. Whereas CNCs are a type of nanorod that is characterized by their high rigidity and crystalline structure. They can be derived from natural cellulose fibers through a process of acid hydrolysis, which removes the less ordered amorphous portions, resulting in 100% cellulose chemical composition in crystalline regions [17].

The extraction of cellulose nanofibers from natural sources typically follows a systematic top-down approach, focusing on isolating lignocellulosic fibers and removing non-cellulosic components through mechanical, chemical, and enzymatic treatments [18]. Initially, raw materials such as plant fibers, corn stalks, or sugarcane bagasse undergo pretreatment steps to remove lignin, hemicellulose, and other impurities. This is followed by mechanical disintegration techniques like high-pressure homogenization or ultrasonication to reduce the cellulose fiber size. Acid hydrolysis, often using sulfuric acid, is then employed to selectively degrade the amorphous regions while preserving the crystalline domains, yielding CNCs. The resulting nanocellulose is thoroughly washed, neutralized, and purified to remove residual chemicals before being dried or functionalized for enhanced compatibility with polymer matrices such as polylactic acid (PLA) [19,20]. The functions of the three main processes, including grinding, alkali extraction, and bleaching, are as follows.

(a) Grinding: Biomass is mechanically ground to increase the accessibility and exposure of cellulose fibers, facilitating subsequent processing. The process breaks the material into particles containing both crystalline and amorphous regions.
(b) Alkali Extraction: The fibers are treated with a sodium hydroxide solution to remove soluble polysaccharides and other non-cellulosic components. This is followed by thorough washing to ensure the fibers are free of residual chemicals.
(c) Bleaching: The material is treated with a sodium chlorite solution under mechanical stirring. This process removes lignin, polyphenols, and proteins, leaving behind purified nanocellulose.

These extraction methods rely on chemical, mechanical, or combined approaches to prepare nanomaterials tailored for specific applications. The main goal during the production process is to preserve the crystalline structure and maintain the polymerization level of the CNF filaments as much as possible. The choice of method often depends on the type of cellulose source, as different sources require distinct processing techniques to achieve optimal yields and properties.

Cellulose exists in different forms, with alpha (α)-cellulose and beta (β)-cellulose being two of the most commonly studied [21]. α-Cellulose is a highly crystalline and stable form, consisting of long-chain cellulose molecules that provide superior mechanical strength, chemical resistance, and thermal stability. Due to its high degree of polymerization and ordered structure, α-cellulose is the preferred form for high-performance applications, such as in reinforced composites, textiles, and biomedical materials [22]. On the other hand, β-cellulose consists of shorter, more amorphous cellulose chains, making it less crystalline and more soluble in alkaline solutions. This lower degree of polymerization allows for easier extraction and processing, making β-cellulose more suitable for applications where flexibility, solubility, or chemical modification is required. However, its reduced structural integrity limits its mechanical performance compared to α-cellulose [23].

### 2.2. Chemical, Mechanical, and Enzymatic Processing Techniques

The preparation of CNFs often involves chemical, mechanical, or enzymatic processes tailored to the source material. Cotton, a commonly used cellulose source, undergoes a chemically intensive process to extract CNFs. The process begins with treating cotton fibers with an acidified sodium chlorite solution to remove lignin, a structural polymer in plant cell walls. The fibers are then washed with distilled water. Subsequently, hemicelluloses are removed using a potassium hydroxide solution, followed by another washing step. Finally, the fibers are treated with hydrochloric acid to ensure thorough purification before a final wash. After the chemical treatment, the cotton fibers are subjected to a short mechanical process, wherein they are ground using a high-speed grinder with a grinding stone rotating at 1800 rpm [24]. Table 1 provides an overview of the key processing techniques of cellulose from cotton and wood pulp sources, including their steps and details.

Wood pulp, a prominent cellulose source, undergoes a primarily mechanical process to isolate CNFs. Wood-based CNFs are highly valued due to their high cellulose purity, strong inter-fiber bonding, superior intrinsic physical properties, negligible axial thermal expansion, and biodegradability [25]. However, their complex structure necessitates intensive mechanical processes for extraction. Researchers at Gorgan University of Agricultural Sciences and Natural Resources [26] demonstrated one example of such a process. They used Paulownia wood particles, which were ground and screened to achieve a particle size corresponding to a 40-mesh screen (420 μm pore size). These wood microparticles were immersed in distilled water for five hours and then processed using a disk grinder at a 2 wt.% concentration. The particles were passed through the grinder six times, with a gradual decrease in particle size from +200 μm to −600 μm, and a stepwise reduction in rotor speed from 2800 to 1800 rpm. This purely mechanical method highlights the advantages of wood pulp as a cellulose source, including its high availability and reduced environmental impact due to the absence of chemical treatments. The versatility of processing techniques ensures that CNFs from various sources can be prepared to meet specific application needs, with each method balancing efficiency, resource availability, and environmental considerations.

### 2.3. Functionalization and Surface Modification Strategies

Surface modification of nanomaterials like CNFs is critical in enhancing their properties for specific applications. By tailoring the surface characteristics, nanocomposites can achieve enhanced electrical conductivity, mechanical strength, and hydrophobicity [27]. Among the many functionalization techniques, acetylation and silylation are two prominent methods for improving the performance and compatibility of CNFs with various matrices.

Acetylation involves introducing acetyl groups to the surface of nanocellulose. This process typically employs a mixture of acetic anhydride (Ac_2_O, as an acyl donor) and dry acetic acid, with a small amount of sulfuric or perchloric acid serving as a catalyst [28]. Acetylation effectively reduces the hydroxyl group density on the nanocellulose surface, enhancing hydrophobicity and compatibility with non-polar polymers. This modification can also improve the dispersion of CNFs in hydrophobic matrices, expanding their utility in composite materials.

Silylation, on the other hand, introduces silyl groups to the nanocellulose surface, significantly improving its compatibility with non-polar matrices. The process involves substituting hydroxyl groups with silyl groups, which can be tuned to achieve varying levels of silylation. At high degrees of silylation (DS > 1), the nanocellulose core chains become soluble in the reaction medium. This results in the disintegration of cellulose crystals and a loss of the original morphology, which may be advantageous for specific applications requiring soluble or highly modified nanocellulose [28].

The choice of surface modification technique—whether acetylation, silylation, or other methods—depends on the desired properties and applications of the nanocomposite. For instance, acetylation may be preferable for applications requiring enhanced hydrophobicity and mechanical performance, while silylation is better suited for compatibility with non-polar systems. As research advances, the development of novel functionalization strategies continues to expand the versatility and performance of CNF-based materials in diverse fields.

### 2.4. Scale-Up Challenges and Industrial Production

Nanofibers have attracted significant interest from researchers and engineers due to their exceptional properties, including a high specific surface area, flexibility, and multi-functionality. These attributes make nanofibers highly suitable for diverse applications, such as insulation, composite material reinforcement, filtration, conductive materials, sensors, drug delivery systems, wound healing, and tissue engineering. Additionally, advanced materials and structures, particularly those incorporating nanotubes, benefit greatly from nanofiber integration [29]. However, despite their immense potential, the widespread industrial adoption of nanofibers is hindered by challenges associated with scaling up production. Achieving uniformity, consistency, and cost efficiency in manufacturing remains a significant hurdle. These issues complicate the use of nanofibers in the rapidly growing field of nanotechnology [30]. Several production methods, including electrospinning, centrifugal spinning, and melt spinning, are employed to manufacture nanofibers, yet each comes with unique technical and scalability challenges.

Electrospinning, a widely used technique, relies on electrical forces to create nanofibers. The setup typically includes a high-voltage supply, a collecting plate, a syringe with a pump, and a polymer solution. The high voltage applied to the tip of the syringe nozzle causes the polymer solution to elongate into a conical shape and produce nanofibers [31]. However, electrospinning faces several limitations that impede its scalability. These include a reliance on high voltage, susceptibility to needle clogging, and low fiber production efficiency. Needleless electrospinning offers some improvement by reducing clogging and increasing yield, but overall production rates remain low compared to alternative methods [30,31]. The inherent limitations of electrospinning—particularly its dependence on high voltage and the challenges of scaling up—have prompted the exploration of other manufacturing techniques.

Centrifugal spinning addresses some of the limitations of electrospinning using centrifugal force instead of electrical forces. In this method, a polymer solution is rotated at high speeds, causing it to be expelled as elongated jets. As the solvent evaporates, the polymer solidifies into nanofibers [30]. This technique is more cost-effective and avoids the need for high-voltage equipment. However, scaling up centrifugal spinning introduces its own challenges, such as maintaining precise control over parameters like temperature and rotational speed. Variations in these parameters can lead to defects such as uneven fiber distribution or inconsistent fiber diameters, complicating large-scale production.

Melt spinning, another promising alternative, relies on centrifugal force to produce nanofibers from molten polymers. The process is highly efficient and suitable for high-throughput production, making it attractive for industrial applications. However, it is not without challenges. Achieving uniform fiber morphology at high rotational speeds is difficult, as increased speed can lead to bulking and misalignment of fibers [32]. Temperature control is critical in melt spinning, as precise regulation is required to maintain the polymer’s optimal viscosity. Fluctuations in temperature can result in polymer degradation, inconsistent fiber diameters, or structural defects, significantly affecting product quality. These challenges become more pronounced in large-scale production, necessitating meticulous optimization of processing parameters.

Overcoming these scale-up challenges is essential to unlocking the full potential of nanofibers in industrial applications. Advances in process control, innovative manufacturing techniques, and a better understanding of material behavior under varying conditions will ensure consistent quality and cost-effective production of nanofibers at scale.

## 3. Properties of Cellulose Nanofibers and Nanocomposites

Cellulose materials exhibit unique physicochemical properties that contribute to their versatility in various applications. The high crystallinity and extensive hydrogen bonding network of cellulose provide excellent mechanical strength, rigidity, and thermal stability [33]. Additionally, the chemical functionality of cellulose allows for modifications, which can enhance the compatibility of cellulose with different matrices. These inherent characteristics of cellulose materials extend to CNFs, which possess exceptional properties that make them highly suitable as reinforcing fillers in composite manufacturing. Their intrinsic strength, stiffness, and durability underscore their potential as an ideal reinforcement material [34]. Beyond their remarkable structural and mechanical characteristics, CNFs exhibit notable thermal stability and excellent barrier properties, enhancing their versatility across various applications [35]. The optical and electrical properties of CNFs, including their transparency and high electrical resistance, further extend their utility to renewable energy technologies, complementing their role in composite reinforcement. Moreover, as a material derived from natural sources, CNFs stand out for their renewability and environmental benefits, making them an eco-friendly choice for sustainable manufacturing practices and renewable energy applications.

### 3.1. Structural and Mechanical Properties

**Figure 1:**
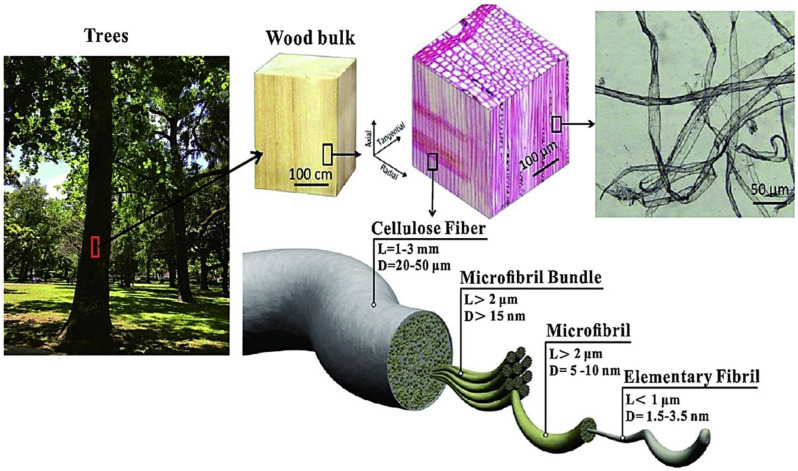
Hierarchical structure of cellulose nanofibers derived from a pine tree. Reproduced with permission from [39].

CNFs exhibit exceptional structural and mechanical properties attributed to their high aspect ratio, crystallinity, and the inherent strength of cellulose. CNFs demonstrate tensile strength comparable to some synthetic fibers, ranging from 1 to 3 GPa, depending on their source and production methods [36]. With a modulus of elasticity often exceeding 114 GPa, they are well suited for reinforcing various composite materials [37]. When incorporated into polymeric or other matrix materials, CNFs significantly enhance the mechanical performance of cellulose-based nanocomposites by improving strength, stiffness, and durability. This enhancement is largely due to the nanoscale size and large surface area of CNFs, which create strong interfacial interactions with the matrix, effectively transmitting stress and increasing load-bearing capacity [38]. Figure 1 illustrates the hierarchical structure of cellulose nanofibers derived from wood, tracing their journey from the macroscopic tree structure to the nanoscale fibers and displaying their highly organized plant cell wall composition, contributing to their mechanical potential.

The mechanical performance of CNF-based composites is influenced by factors such as fiber orientation, distribution within the matrix, and the degree of fiber–matrix bonding. Uniform dispersion and alignment of CNFs are critical for ensuring consistent stress distribution and minimizing weak points in the composite. Conversely, poor dispersion can lead to fiber clumping or agglomeration, which diminishes material strength and overall performance [40]. Techniques such as chemical surface modifications and compatibilizers are commonly employed to enhance fiber–matrix interactions, thereby improving the uniformity and reliability of the composite’s properties.

Additionally, CNF-reinforced composites exhibit outstanding fatigue resistance, making them ideal for applications that involve repeated stress or strain over extended periods. CNFs’ ability to maintain mechanical integrity under cyclic loading conditions offers a significant advantage in industries such as aerospace and automotive, where durability and safety are paramount [41]. Furthermore, these nanocomposites provide lightweight alternatives to conventional materials, meeting the demands for weight reduction without compromising strength, particularly in industries where both efficiency and performance are critical [37].

Apart from this, CNFs, characterized by their small diameters and three-dimensional interconnected fiber networks, coupled with their renewable and biodegradable nature and high chemical and thermal stability, are highly effective for dye removal from wastewater [42]. Khatri et al. [43] have explored the fabrication of reusable, eco-friendly electrospun cellulose acetate (CA)/CNC nanofibers for efficient methylene blue dye removal from wastewater. The developed nanofibers demonstrate high adsorption capacity, stability, and recyclability, making them a promising sustainable solution for water purification.

### 3.2. Thermal Stability and Barrier Properties

Beyond their mechanical advantages, CNFs and CNF-based nanocomposites exhibit excellent thermal stability and barrier properties, making them highly suitable for applications in packaging and insulation. CNFs demonstrate moderate thermal stability, with thermal degradation typically occurring at around 200 °C, depending on their purity and production conditions [44]. When incorporated into nanocomposites, their thermal stability can be enhanced further depending on the selected matrix material. For example, integrating CNFs into thermoplastics like PLA or polyethylene can increase the composite’s heat resistance by 5–15 °C, effectively delaying premature degradation [45]. Understanding the thermal properties of these materials is crucial for researchers to evaluate the potential of nanocomposites for various applications. Dynamic mechanical analysis (DMA) and thermomechanical analysis (TMA) are key techniques used to assess the thermomechanical properties of nanocomposites. DMA is commonly employed in nanocomposite characterization as it enables the analysis of viscoelastic properties of material across a wide temperature range [37]. TMA, on the other hand, evaluates dimensional stability, thermal expansion, and mechanical behavior under temperature variations. Thermogravimetric analysis (TGA) is used to assess the thermal stability and degradation of nanocellulose composites by measuring weight loss at different temperatures to help understand the resistance of the material to heat and its decomposition patterns. Differential scanning calorimetry (DSC), on the other hand, analyzes thermal transitions such as the glass transition temperature, crystallization, and melting points, offering valuable information about the thermal behavior, crystallinity, and compatibility of material with various polymer matrices [46].

One of the most significant advantages of CNFs is their exceptional barrier properties against gases such as oxygen and moisture, making them ideal for food packaging applications. The dense network of cellulose fibers hinders gas permeation, ensuring better preservation of consumable goods [36]. These characteristics position CNF-based materials as a sustainable alternative to traditional plastic packaging.

The synergy of thermal resistance and superior barrier properties in CNF-based nanocomposites makes them a promising option for replacing conventional insulating materials. Researchers are actively exploring hybrid nanocomposites by incorporating other nanomaterials, such as graphene or clay, into CNF matrices. These hybrids further enhance thermal stability and barrier performance, unlocking new opportunities in energy storage, flexible electronics, and eco-friendly packaging solutions [41].

### 3.3. Optical and Electrical Characteristics

CNFs exhibit remarkable optical and electrical properties, making them versatile materials for applications in electronics, renewable energy, pharmaceuticals, and advanced building materials [47]. One of the most striking optical characteristics of CNFs is their transparency, which stems from their nanoscale dimensions being smaller than the wavelength of visible light. This allows CNF films to transmit light with minimal scattering, making them suitable for applications requiring high transparency, such as flexible electronics, displays, solar cells, and food packaging [48]. Another key optical property is birefringence: the variation in refractive indices along different axes. This phenomenon, influenced by the relative proportions of crystalline and amorphous regions in the fibers, provides valuable insights into the molecular orientation and structure of CNFs [49]. Cellulose nanomaterials have the ability to spontaneously organize into chiral nematic structures, which leads to birefringence and distinctive optical characteristics. This arrangement also causes incident light to become circularly polarized. These unique properties open up opportunities for using cellulose-based materials in various applications, including encryption and sensing technologies [50]. The refractive index of CNF films is highly tunable, depending on factors such as crystallinity and the interaction of light within the fiber matrix, enabling precise tailoring for optical applications. CNFs also exhibit thermoresponsive behavior when combined with thermochromic pigments. These hybrid films can modulate light transmission, dropping to as low as 4% at specific temperatures while regaining up to 60% transparency when heated above a threshold [51]. Such properties are ideal for smart windows and adaptive optical materials, enabling energy-efficient light management. Cellulose-based materials are highly sensitive to environmental factors like pH, humidity, and temperature, which makes them well suited for luminescent sensing applications. They have also been applied in areas like encryption, bioimaging, and analytical tools. However, there are still many aspects of this field that remain unexplored [50].

In the realm of electronics and energy storage, CNFs stand out for their unique electrical characteristics. As an insulator with high electrical resistance due to sp3-hybridized carbons in cellulose molecules, CNFs initially present challenges for electron flow. However, these limitations are effectively overcome when CNFs are combined with conductive materials like carbon nanotubes, MXenes, or metallic nanoparticles. The resulting CNF-based composites exhibit enhanced electrical conductivity and thermal stability, making them suitable for advanced energy applications such as batteries and supercapacitors [52]. The porous structure of CNFs also promotes efficient ion transport, significantly boosting the energy storage capacity of flexible supercapacitors and electrochemical devices [53]. Furthermore, CNF composites balance high mechanical strength and flexibility, which are critical for wearable electronics and other applications requiring robust yet adaptable materials.

Research into CNFs continues to unlock their potential in diverse fields. For instance, CNFs are being explored for electromagnetic interference (EMI) shielding, electric heating systems, and other energy-related technologies [52]. By serving as a sustainable scaffold, CNFs enable the construction of lightweight, high-performance electrodes and other advanced components for renewable energy systems.

Table 2 highlights the versatility of CNFs across diverse industries, emphasizing their role as a sustainable and high-performance material in advancing green technologies and innovative solutions. The combination of sustainability, optical clarity, and versatile electrical performance positions CNFs as a transformative material in pursuing greener and more efficient technologies across multiple industries.

### 3.4. Biocompatibility and Environmental Impact

CNFs have garnered significant attention due to their exceptional mechanical properties, inherent biocompatibility, and sustainability. Sourced predominantly from plants, CNFs are emerging as versatile materials for applications in biomedical devices, packaging, and electronics, often serving as eco-friendly alternatives to conventional synthetic materials [54].

The biocompatibility of CNFs is rooted in their natural origin. As a primary component of plant cell walls, cellulose is inherently compatible with biological systems. The nanoscale dimensions of CNFs enhance their surface area, making them particularly suited for applications in tissue engineering, wound healing, and drug delivery systems. Studies have shown that CNF scaffolds promote cell adhesion and proliferation, making them ideal candidates for regenerative medicine [55]. However, the biocompatibility of CNFs can be influenced by the methods used during their processing. Chemical modifications, often employed to improve mechanical properties or tailor surface functionalities, can alter the fibers’ surface chemistry, potentially affecting cell interaction [56]. As such, maintaining or enhancing biocompatibility requires careful selection of processing techniques and surface treatments.

CNFs are renewable and biodegradable materials with a markedly lower environmental footprint than synthetic polymers. Their production typically involves mechanical or chemical biomass processing, and when sourced from sustainably managed forests or agricultural residues, CNFs offer significant environmental advantages. By replacing plastics and other non-biodegradable materials in applications like packaging and filtration systems, CNFs reduce the global reliance on fossil fuel-derived materials [54,57]. Despite their eco-friendly potential, challenges remain in scaling up CNF production. Chemical treatments, particularly those involving harsh acids or solvents, can lead to environmental concerns if waste streams are not adequately managed. Innovations in green chemistry are addressing these issues, emphasizing enzyme-assisted extraction techniques and using less toxic reagents to minimize ecological impact [58].

CNFs’ biodegradability is a critical advantage in advancing circular economy goals. Unlike synthetic polymers, CNFs naturally degrade under appropriate environmental conditions without releasing harmful byproducts [59]. They can be composted at the end of their lifecycle, providing a sustainable disposal pathway that aligns with efforts to reduce landfill waste. Research confirms that cellulose-based materials exhibit efficient biodegradation, making them an ideal substitute for traditional plastics in applications demanding environmental responsibility. Overall, CNFs’ combination of biocompatibility, sustainability, and biodegradability positions them as a transformative material in fostering greener, more sustainable industries. Continued advancements in eco-friendly production methods and careful consideration of surface modifications will expand their potential across diverse applications.

## 4. Nanocomposites Based on Cellulose Nanofibers

Nanocomposites that incorporate CNFs represent a significant advancement in materials science, offering a unique combination of enhanced mechanical, thermal, and barrier properties. As a reinforcing agent, CNFs contribute to the development of materials that are not only lightweight and robust but also environmentally sustainable. By integrating CNFs into various polymer matrices, researchers have been able to engineer eco-friendly alternatives to conventional composites, which are typically derived from non-renewable fossil fuels and lack biodegradability [60]. These nanocomposites leverage the nanoscale dimensions and high aspect ratio of CNFs to improve matrix interactions, leading to superior performance characteristics. The resulting materials find applications in diverse industries, including automotive, aerospace, packaging, and biomedical engineering. Among biodegradable polymers, PLA stands out as one of the most promising options due to its excellent biocompatibility, biodegradability, optical clarity, and widespread availability at a cost-effective price [61]. Over the past few decades, PLA has been extensively researched for use in packaging, textiles, orthopedics, tissue regeneration, drug delivery, implants, and medical instruments [62]. It has been observed that PLA bio-nano composites reinforced with crystalline nanocellulose exhibit excellent mechanical, thermal, and physical properties [63]. Furthermore, the addition of CNCs to the PLA matrix is found to improve the crystallinity because it acts as a nucleating agent [64]. This enhancement in crystallinity improves the mechanical properties and performance of the composite.

The mechanical performance of nanocellulose composites is significantly influenced by the type, concentration, and dispersion of nanocellulose within the matrix. Nanocellulose, including CNCs and CNFs, acts as a reinforcing agent, improving tensile strength, Young’s modulus, and impact resistance due to its high aspect ratio, superior stiffness, and strong interfacial interactions with the matrix [65]. However, the efficiency of reinforcement depends on achieving uniform dispersion and strong interfacial adhesion between the nanocellulose and the matrix. The mechanical properties of nanocellulose are largely determined by its structural morphology. Key factors that influence these properties include the cellulose source, density, degree of polymerization, aspect ratio, crystallinity, fibril alignment, and any surface modifications applied. These elements collectively play a crucial role in shaping the overall performance and behavior of nanocellulose in various applications. The surface characterization of nanocellulose films and coatings can reveal key insights into their mechanical and structural properties. Topographical and microscopic evaluations provide coating porosity and density, while FTIR spectra demonstrate the relationships with hydrogen bonding energy [66].

### 4.1. Reinforcement Mechanisms and Matrix Interaction

The reinforcement mechanisms of CNFs in nanocomposites are primarily governed by mechanical interlocking and hydrogen bonding, which significantly enhance the mechanical properties of the composites [67]. The high aspect ratio of CNFs enables the formation of entangled networks within the polymer matrix [68]. This network structure enhances load-transfer efficiency, improving the composite’s strength and stiffness [69].

A crucial contributor to reinforcement is the hydrogen bonding between the hydroxyl groups on the CNF surface and the polymer matrix. These interactions strengthen the fiber–matrix interface, facilitating effective stress transfer during mechanical loading. As a result, CNF-reinforced composites exhibit superior tensile strength, Young’s modulus, and impact resistance compared to composites with conventional fillers like carbon black or glass fibers [70].

The orientation of CNFs within the matrix further influences composite performance. Aligned CNFs can significantly enhance mechanical properties along specific directions. Techniques such as electrospinning and magnetic alignment are employed to achieve anisotropic properties, where the composite exhibits tailored characteristics based on directional stresses. Such alignment strategies make CNF-based composites adaptable for specialized applications requiring directional strength.

CNFs are inherently hydrophilic, making their dispersion in hydrophobic polymer matrices, such as polypropylene or polystyrene, challenging. To address this, surface modifications are often used to enhance compatibility.

1. Chemical Surface Modification: Hydroxyl groups on CNFs can be functionalized with organic molecules or grafted with hydrophobic chains. This improves dispersion within hydrophobic matrices and enhances interfacial adhesion, thereby boosting the mechanical properties of the resulting nanocomposites [71].
2. Hydrophilic Matrix Integration: Blending CNFs with hydrophilic polymers like polyvinyl alcohol (PVA) or polylactic acid (PLA) can circumvent surface modification. These polymers naturally bond with CNFs, forming strong interfacial interactions. Additionally, using biodegradable matrices like PLA in combination with CNFs results in fully sustainable composites that are high-performing and environmentally friendly [72].

CNF-reinforced nanocomposites find applications across various industries due to their exceptional properties and sustainability: (1) Automotive Sector: CNF-reinforced polymers reduce vehicle weight while maintaining structural integrity, leading to improved fuel efficiency and lower carbon emissions [73]. (2) Packaging Industry: CNF-based nanocomposites offer excellent barrier properties against moisture and gases, making them ideal for food packaging. These properties help extend shelf life and minimize spoilage, contributing to sustainable packaging solutions [74]. (3) Biomedical Field: The biocompatibility and mechanical strength of CNF nanocomposites make them suitable for prosthetics, implants, and drug delivery systems. Functionalization with bioactive molecules further expands their potential for targeted drug delivery and tissue engineering.

The interaction between CNFs and the polymer matrix is central to the performance of nanocomposites. Through mechanical interlocking, hydrogen bonding, and tailored surface modifications, CNF-reinforced composites offer a blend of strength, sustainability, and versatility. These features position them as promising materials for advanced applications across diverse sectors, paving the way for innovations in lightweight structures, eco-friendly packaging, and biomedical solutions.

### 4.2. Fabrication Techniques for Nanocomposites

Nanocomposites, composed of nanoparticles embedded within a matrix, are engineered to enhance properties such as mechanical strength, thermal stability, and electrical conductivity. The fabrication methods for these materials include phase separation, template-assisted synthesis, 3D printing, and solution casting, each offering unique advantages and customization opportunities [75].

#### 4.2.1. Phase Separation

**Figure 2:**
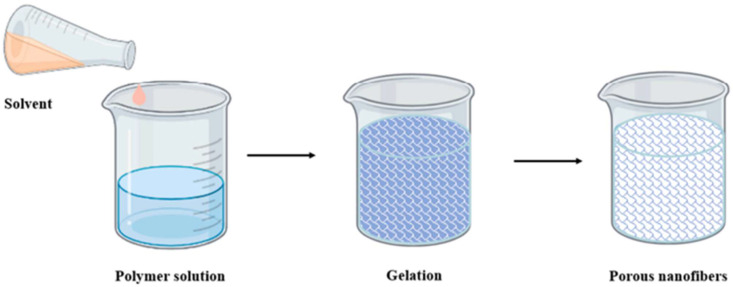
Nanofiber generation by phase separation. Reproduced with permission from [75].

Phase separation is widely used for fabricating nanocomposites, particularly nanofibers. This technique begins with a homogeneous polymer solution prepared using a solvent like tetrahydrofuran (THF) [75]. The separation of the polymer and solvent into distinct phases is induced by adding a nonsolvent or applying thermal treatment, leading to gelation. Gelation, a critical step, determines the porosity and structure of the composite. After gelation, the material undergoes freezing and freeze-drying to remove the solvent, yielding the final structure. Known as solid–liquid phase separation or ice segregation-induced self-assembly, this method offers precise control over the internal structure of the nanocomposite. By modifying parameters such as solvent type, temperature, or polymer concentration, the size, distribution, and orientation of nanoparticle domains can be tailored to enhance mechanical strength, electrical conductivity, or thermal stability. This cost-effective and versatile technique is widely used for producing nanocomposites with specific structural and functional properties. Figure 2 shows the steps of the phase separation process.

#### 4.2.2. Template-Assisted Synthesis

Template-assisted synthesis is an advanced fabrication method that utilizes hard or soft templates to shape materials. Hard templates are often used to produce nanotubes, while soft templates are ideal for wire-like structures [75]. A notable advantage of this approach is its ability to create flexible materials, although the labor-intensive process of template removal—requiring dissolution or calcination—remains a limitation. This technique is especially effective for synthesizing hierarchical nanofibers and enhancing the electrochemical performance of metal oxide electrode materials. Transition metal oxides (TMOs) are a prominent focus due to their excellent properties and industrial applications, with template-assisted synthesis enabling precise control of their morphology and physicochemical characteristics [76]. Soft templates, including block copolymers and biological materials, are favored for their ease of removal compared to hard templates. Additionally, template-assisted synthesis has been instrumental in fabricating porous carbons for supercapacitor applications, allowing for adjustable pore size distribution and specific structural designs to meet functional requirements.

#### 4.2.3. 3D Printing

3D printing has emerged as a transformative technology for nanocomposite fabrication, offering advantages such as material customization, enhanced integration, and efficient prototyping. By incorporating nanomaterials into polymer matrices, this method improves mechanical strength and functional properties. Nanomaterials can be added in two ways: either by pre-mixing them into the polymer matrix before printing or during pauses in the printing process for manual or automated insertion. This versatility enables the creation of complex, high-performance nanocomposite structures with tailored properties, expanding the applications of nanomaterials in additive manufacturing [77]. However, the challenges associated with printing nanocellulose structures such as collapsing and shape fidelity or retention during printing and drying need to be addressed to implement this process effectively [78].

#### 4.2.4. Solution Casting

Solution casting involves dissolving a polymer matrix in a solvent that can swell clay particles, facilitating the incorporation of nanoparticles. The mixture is stirred mechanically or ultrasonically to ensure the polymer chains penetrate the clay galleries, achieving a uniform dispersion of nanoparticles. This technique is particularly effective for polymers with low or no polarity, as it avoids degradation of both the polymer and any organic modifiers under high temperatures [79]. Once the nanoparticles are evenly dispersed, the solution is cast into molds or onto surfaces, and the solvent is allowed to evaporate, leaving a solid nanocomposite film. The resulting material exhibits enhanced properties due to the uniform distribution of nanoparticles, making solution casting a reliable and versatile method for fabricating polymer-based nanocomposites.

Each fabrication technique offers unique benefits and caters to specific requirements in nanocomposite production. Phase separation enables precise control of internal structures, template-assisted synthesis tailors material morphology, 3D printing provides customization and efficiency, and solution casting ensures uniform nanoparticle dispersion. Together, these methods drive advancements in nanocomposite technology, enabling their application across diverse fields such as energy storage, electronics, automotive, and biomedical engineering.

### 4.3. Tailoring Properties for Specific Applications

CNFs are gaining prominence across various industries due to their exceptional properties, including a high surface area, biodegradability, and remarkable mechanical strength. Researchers are actively exploring ways to tailor the properties of CNFs to optimize their performance for specific applications. A central focus is on modifying the surface properties of CNFs to enhance their compatibility with diverse polymer matrices, which is a critical factor in creating efficient composite materials.

Surface modification is a widely employed approach to improving the interaction of CNFs with polymer matrices. This process often involves introducing functional groups onto the CNF surface through chemical treatments such as esterification or acetylation. These modifications significantly enhance the dispersion and mechanical performance of CNFs within the composite material. For instance, when surface-modified CNFs are incorporated into polymers like PLA or polyethylene (PE), they exhibit notable improvements in tensile strength and thermal stability [80]. These enhancements make surface-modified CNFs an attractive option for high-performance applications, ranging from automotive components to sustainable packaging solutions.

Mechanical processing techniques also play a crucial role in customizing the characteristics of CNFs. High-pressure homogenization is one such method that reduces the diameter of CNFs, thereby increasing their aspect ratio. A higher aspect ratio not only improves the mechanical properties of the resulting composites but also ensures they remain lightweight. Additionally, mechanical treatments contribute to a uniform distribution of CNFs within the polymer matrix, which is essential for maximizing composite performance. This uniformity is particularly advantageous in applications such as packaging materials, where consistent strength and durability are critical, and in construction materials, where lightweight yet robust composites are highly valued.

Tailoring CNFs for specific applications involves careful selection of polymer matrices and deliberate modification of CNF properties. This approach enables the development of composites with targeted characteristics suited for specific industries. For example, in the production of biodegradable packaging, CNFs can be blended with starch-based polymers to enhance strength and moisture resistance without compromising biodegradability [81]. Similarly, for biomedical devices, CNFs can be incorporated into biocompatible polymers to improve mechanical strength and thermal stability while maintaining their biocompatibility. In electronics, CNFs can serve as lightweight, conductive reinforcements, enabling the fabrication of flexible, sustainable components.

By leveraging surface modification, mechanical processing, and targeted material design, cellulose nanofibers can be precisely tailored to meet the demands of diverse applications. From packaging and construction to biomedical and electronic devices, CNFs offer an environmentally friendly and versatile solution, underscoring their potential as a transformative material in modern industries.

### 4.4. Challenges in Achieving Homogeneous Dispersions

Achieving homogeneous dispersions of CNFs in various matrices is crucial for optimizing their performance in composites and coatings. However, this remains a significant challenge due to CNFs’ intrinsic tendency to aggregate, driven by strong hydrogen bonding and van der Waals forces. Such agglomeration leads to uneven distribution within the matrix, adversely affecting the mechanical properties and overall functionality of the resulting materials. A comprehensive discussion on the dispersion properties of nanocellulose can be found in [82].

To address aggregation, researchers have explored mechanical methods such as ultrasonication and high-shear mixing. These techniques effectively break down agglomerates, promoting finer dispersion of CNFs within the matrix [83]. While these approaches have shown promise, achieving uniform dispersion often requires fine-tuning processing parameters, such as the duration and intensity of ultrasonication or mixing.

Chemical treatments offer an additional avenue for improving dispersion. Surfactants, for instance, can reduce the surface tension between CNFs and the surrounding matrix, stabilizing the dispersion and preventing re-agglomeration during processing [83]. The selection of appropriate solvents and optimization of processing conditions, including temperature and shear rates, further enhance the effectiveness of dispersion methods, enabling better scalability from laboratory to industrial applications.

A significant challenge lies in ensuring compatibility between CNFs and the polymer matrices into which they are incorporated. Poor compatibility can result in weak interfacial bonding and phase separation, diminishing the mechanical performance of the composite [84]. Surface modification of CNFs, such as grafting functional groups onto their surface, can enhance compatibility and promote better interfacial adhesion with the matrix material [82]. This not only improves dispersion but also reinforces the mechanical integrity and stability of the composite.

Homogeneous dispersions that are relatively easy to achieve at the laboratory scale often present significant difficulties during industrial-scale production. Variations in mixing equipment, batch sizes, and processing conditions can lead to inconsistent dispersion quality. Developing robust methods and scalable technologies that ensure uniform dispersion across large quantities is essential for transitioning CNF-based composites to commercial applications.

The rheological properties of CNF dispersions can pose additional challenges during processing. High-viscosity dispersions, common in CNF systems, can hinder flow and mixing, making it difficult to achieve uniform distribution within the matrix. Researchers are investigating the use of surfactants, dispersants, and other additives to modify the rheological behavior of CNF dispersions, enabling smoother processing and ensuring homogeneous dispersion in the final product [85].

While significant progress has been made in dispersing cellulose nanofibers uniformly, challenges remain in overcoming aggregation, ensuring compatibility with polymer matrices, scaling up production, and addressing rheological issues. Continued research into advanced mechanical techniques, chemical treatments, and scalable processes is vital to fully unlock the potential of CNFs in diverse industrial applications.

### 4.5. Life Cycle Assessment of CNFs

As a renewable and biodegradable material, CNFs offer a lower carbon footprint compared to synthetic nanomaterials like carbon nanotubes and traditional polymers. Their production relies on biomass sources, reducing reliance on fossil fuels and mitigating environmental pollution. However, the extraction and processing of CNFs, which typically involve mechanical, chemical, and enzymatic methods, can be energy-intensive and require significant water usage. This may raise concerns regarding resource efficiency and environmental impact. Chemical treatments, such as alkali extraction and bleaching, can also result in waste products that require careful management. Despite several challenges, CNFs demonstrate considerable advantages in their end-of-life phase. Unlike many synthetic materials, CNFs decompose naturally, leaving no toxic residues, making them a more sustainable choice over many other materials [86,87].

## 5. Applications of Cellulose Nanofibers, Nanomaterials, and Composites

CNFs and nanomaterials are critical in developing advanced composites across diverse industries. Derived from renewable resources, these sustainable materials offer exceptional mechanical properties, lightweight characteristics, and broad applicability. By enhancing the functionality of conventional materials, cellulose-based nanomaterials not only support environmental sustainability but also enable the development of innovative products with advanced capabilities, driving a transition toward greener and more efficient technologies.

The applications of cellulose-based nanofibers, nanomaterials, and composites can be broadly categorized into high-volume and low-volume uses. High-volume applications typically involve large-scale production and widespread utilization, such as the following: (1) Paper and Packaging Materials: CNFs are used to enhance the strength, flexibility, and barrier properties of paper and packaging products, offering sustainable alternatives to plastics. (2) Automobile Components: CNFs and composites are increasingly incorporated into automotive parts to reduce weight and improve fuel efficiency while maintaining structural integrity. On the other hand, low-volume applications focus on specialized, high-value fields where the unique properties of cellulose nanomaterials are critical, including the following: (3) Aerospace Industry: CNFs and cellulose nanocomposites are used in lightweight, high-strength materials for aircraft and space applications, contributing to improved fuel efficiency and performance. (4) Biomedical and Pharmaceutical Fields: CNFs and CNCs are employed in drug delivery systems, wound dressings, and tissue engineering, where biocompatibility, biodegradability, and mechanical strength are essential [88]. The choice of cellulose-based material—whether cellulose nanofibers (CNFs), cellulose nanocrystals (CNCs), or cellulose nanomaterials (CNMs)—depends on the intended application. For instance, CNFs are favored for applications requiring flexibility and toughness, CNCs are utilized in scenarios demanding high rigidity and strength, and CNMs offer a balance of properties suitable for hybrid or multi-functional composites.

Table 3 highlights a diverse range of high-volume and low-volume applications for CNFs, CNCs, and CNMs. These materials demonstrate unparalleled versatility, serving as a cornerstone for industries striving to balance performance, sustainability, and innovation. By fostering advancements in both established and emerging markets, CNFs and nanomaterials are poised to become integral to next-generation technologies, enabling products that are not only high-performing but also environmentally responsible.

### 5.1. Use in Packaging, Automotive, and Aerospace Industries

CNFs are gaining significant attention in the packaging sector for their exceptional barrier properties, which enhance the shelf life of food products while offering a sustainable alternative to petroleum-based materials. By reducing reliance on plastics, CNFs contribute to minimizing plastic waste. Their lightweight and high-strength characteristics make them ideal for creating sustainable packaging solutions without compromising performance.

A prominent application of cellulose nanomaterials in packaging involves replacing traditional fiber and plastic packaging. As renewable and biodegradable materials derived from cellulose, they reduce the use of non-biodegradable components commonly discarded in packaging waste. For instance, nanomaterial-based foams are being tested as alternatives to traditional polystyrene foams. These foams benefit from CNF reinforcement, which strengthens the foam cells, offering a lightweight, renewable option that replaces polymers derived from fossil fuels [88].

Cellulose nanomaterials enhance existing materials, such as paperboard, by increasing the fiber-to-fiber bond strength. This reinforcement improves packaging durability, reduces material weight, and decreases fuel consumption during transportation [88]. Moreover, CNFs serve as greaseproof coatings for paper packaging, reducing porosity and improving the adhesion of wet additives, such as ink. This enables the use of lighter, thinner papers while enhancing resistance to water vapor and oxygen permeability. For example, CNC-coated materials with cross-linked aluminum and iron ions create strong barriers to oxygen, water vapor, oil, and grease, making them viable alternatives to polymer films [89].

**Figure 3:**
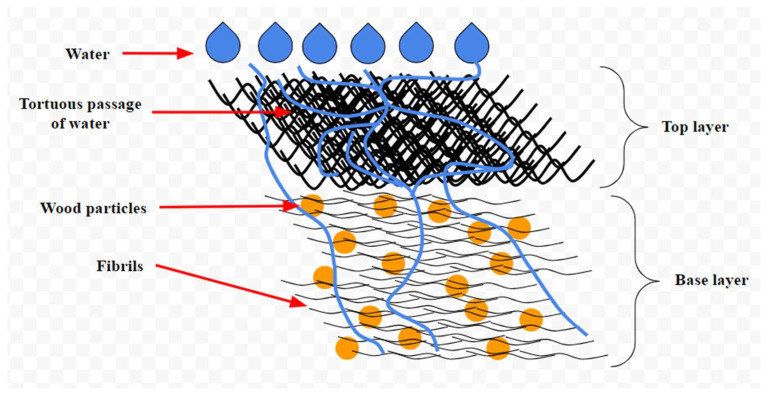
The mechanism of water absorption through the CNC coating is created by cross-linking aluminum and iron ions, creating a stronger barrier against water and grease.

These innovations also make packaging materials more recyclable. Traditional disposable paper food containers often feature plastic coatings that hinder recyclability and may contain harmful substances like polyfluoroalkyl substances (PFAs). In contrast, CNC-coated papers are biodegradable, plant-based, and chemical-free, enabling recycling, and reducing environmental and health concerns [90]. Figure 3 illustrates the process of water absorption in food packaging material coated with a similar CNF layer. While the CNF coating does not entirely prevent water penetration, it significantly alters the water’s path. The layered structure of the coating forces water to take a more tortuous route before reaching the base layer, effectively slowing the flow rate and enhancing the material’s water resistance

The automotive sector is increasingly adopting cellulose nanofibers to develop lighter, more sustainable materials for vehicle components. Incorporating CNFs into composites enables manufacturers to create lightweight yet robust parts that improve fuel efficiency without compromising safety standards. These materials are ideal for producing components such as interior panels, seat structures, and insulation materials [88,91]. CNFs offer high mechanical properties, enhancing stiffness and chemical resistance in automotive components [70,92]. For example, the Ford Motor Company has utilized cellulose nanomaterials to develop body panels and interior trims, benefiting from their cost-effectiveness compared to other composite fibers. The reduced weight of CNF-based components can decrease a vehicle’s weight by up to 340 kg, significantly boosting fuel efficiency. In interior applications, CNFs blended with polymers such as polyethylene and polypropylene improve mechanical and barrier properties while increasing abrasion resistance. These innovations support the development of lightweight, durable interior trims like dashboards and door panels [88].

In aerospace, where the demand for lightweight yet strong materials is paramount, CNFs present a promising solution. Their high strength-to-weight ratio and ability to withstand extreme conditions align with the industry’s sustainability goals. CNFs are being explored for composite materials used in structural components, enabling significant weight and fuel savings in flight operations. Cellulose nanocomposites reinforce exterior aerospace components, improving structural strength, thermal resistance, and electrical conductivity. For interiors, CNF applications in components such as panels, floorboards, and seats reduce overall aircraft weight. Lighter and more efficient designs allow additional seating capacity, reducing greenhouse gas emissions and enhancing fuel efficiency [88].

By leveraging the unique properties of cellulose-based materials, the packaging, automotive, and aerospace industries are advancing toward sustainable and high-performance solutions that address environmental challenges and improve material efficiency.

### 5.2. Applications in Biomedical and Pharmaceutical Fields

The biomedical field represents a promising avenue for using CNFs and their composites. With their biocompatibility and non-toxicity, CNFs are being explored for diverse applications, including drug delivery systems, wound dressings, and tissue engineering scaffolds.

In drug delivery, cellulose-based nanoparticles can encapsulate therapeutic agents, enabling controlled release and targeted delivery [93]. This feature enhances treatment efficacy while minimizing side effects. For example, CNF coatings on drug-loaded matrices stabilize nanoparticles and serve as drug carriers, improving the controlled release of hydrophobic drugs such as Itraconazole. Studies show these systems improve performance by extending drug retention times and ensuring precise dosage [94]. Similarly, CNF-based aerogels have proven effective for hydrophilic drug loading, significantly enhancing the retention time of medications like Bendamustine up to five times compared to traditional formulations [94,95]. CNF hydrogels have also demonstrated the ability to reduce cancer cell migration and adhesion, offering potential in anti-cancer therapies.

Cellulose nanofiber-based dressings provide excellent moisture retention and create a conducive environment for wound healing. Their antimicrobial properties help prevent infections, making them ideal for treating chronic wounds [93]. For instance, integrating silver nanoparticles (AgNPs) into CNFs enhances their antibacterial capabilities. Methods such as chemical reduction or electrostatic interactions allow for the effective incorporation of AgNPs into CNF membranes, reducing tissue damage and infection risks during healing [94,96]. Zinc oxide (ZnO) is another promising agent combined with CNFs for its antimicrobial and biocompatible properties.

The wound healing process involves tissue regeneration through clot formation, macrophage and neutrophil activation, fibroblast activity, and collagen production. However, infections, particularly in burn wounds, can delay this process, leading to severe complications. CNF-based nanomaterials offer effective solutions by managing wound exudates, maintaining a moist environment, and incorporating antimicrobial agents like AgNPs and ZnO to bolster healing outcomes [94].

In tissue engineering, CNFs serve as scaffolds that support cell growth and tissue regeneration, aiding the development of artificial organs and regenerative medicine. For example, CNFs combined with titanium dioxide (TiO_2_) and silver (Ag) nanoparticles exhibit antibacterial and bioactive properties that promote mineralization and bone tissue growth in simulated body fluids at body temperature (~98.6 °F) [93]. Similarly, CNFs derived from cotton fibers through acid hydrolysis have demonstrated the ability to provide structural support for regenerating human tissue. The rough surface of CNF materials enhances cell adhesion, growth, and bone formation, further reinforcing their suitability for tissue engineering applications [97].

In the pharmaceutical industry, CNFs improve drug formulation and stability by enhancing the solubility of poorly soluble drugs, thereby increasing bioavailability. Efficient delivery systems are crucial in modern medicine, and CNFs provide a platform for precise drug administration. For example, CNF matrices stabilize hydrophobic drugs, enabling controlled release and targeted delivery, which is essential for therapeutic efficacy [94]. CNF-based hydrogels and aerogels also offer unique properties like high elasticity, water retention, and low toxicity, making them suitable for advanced pharmaceutical applications, including hydrophilic drug formulations and cancer therapies.

The unique properties of cellulose nanofibers—biocompatibility, non-toxicity, and versatility—position them as valuable materials in biomedical and pharmaceutical fields. Their potential to revolutionize drug delivery, enhance wound healing, and support tissue regeneration highlights their importance in advancing healthcare solutions.

### 5.3. Role in Energy Storage and Environmental Remediation

CNFs are at the forefront of innovations in energy storage and environmental remediation, offering sustainable and high-performance solutions for critical challenges. Energy storage technologies are evolving rapidly, and CNFs have shown great promise in supercapacitors and batteries due to their high surface area, mechanical strength, and conductivity [98,99]. These energy storage devices combine high power density, rapid charge–discharge capabilities, and low maintenance costs, making them ideal for modern power demands. Supercapacitors store energy through electrostatic charges at the electrode–electrolyte interface and often feature hybrid designs incorporating both double-layer and pseudo-capacitive charging mechanisms. CNFs, with their high surface area and eco-friendliness, significantly enhance the performance of flexible supercapacitors, achieving capacitance values up to 410 F at 0.8 A and retaining 93% of total capacitance after 5000 cycles. By contrast, traditional supercapacitors often store only 85.3% after 2000 cycles. When combined with materials like silver nanoparticles and graphene oxide, CNFs further improve charge storage capabilities. Additionally, using recycled paper fibers with CNFs in supercapacitor anodes creates environmentally sustainable devices with excellent specific capacitance and cycling stability [98].

CNFs have shown exceptional potential as anodes for sodium-ion batteries. Bacterial cellulose (BC), a nanofiber form of cellulose produced through bacterial fermentation, offers high porosity, biodegradability, and efficient production rates. Its ultrafine nanofiber structure, with diameters of 10 to 50 nm, provides excellent electrochemical performance and large surface area. Incorporating compounds like urea, polypyrrole, and polyaniline into BC introduces nitrogen into the material’s structure, enhancing conductivity and surface reactivity, which are critical for efficient sodium-ion battery performance [99].

CNFs also play a transformative role in environmental remediation, particularly water purification and pollution control. The high surface area and reactivity of CNFs make them highly effective for adsorbing pollutants and heavy metals from wastewater. CNF-based composites can be engineered to target specific contaminants, providing sustainable and efficient solutions for cleaning polluted water. CNMs are versatile and have been used to create adsorbents, aerogels, hydrogels, and membranes for a variety of water purification applications, including the following:

(1) Bacteria Removal: CNFs can filter out bacterial contaminants from water.
(2) Heavy Metal Extraction: CNFs effectively adsorb ions such as lead, cadmium, and arsenic, addressing critical water safety challenges.
(3) Oil–Water Separation: CNFs’ hydrophobic and oleophilic properties enable them to separate oil from water efficiently.
(4) Turbidity Reduction: By removing suspended particles, CNFs can significantly enhance water clarity and quality [100].

CNFs’ biodegradability ensures that these materials can be safely disposed of, minimizing their environmental impact while addressing pressing water and air pollution issues. Thus, cellulose nanofibers offer transformative potential in both energy storage and environmental remediation. By enabling high-efficiency, sustainable solutions for energy demands and pollution challenges, CNFs stand as critical materials for advancing global sustainability initiatives.

### 5.4. Potential in Smart Materials and Responsive Systems

CNFs hold immense promise for advancing smart materials and responsive systems. Researchers are leveraging their unique properties, such as high surface area, mechanical strength, and eco-friendliness, to create materials that respond dynamically to external stimuli like temperature, pH, or light. Functional modifications of CNFs or their integration into polymer matrices enable applications in coatings, textiles, biosensors, and energy-efficient materials. For example, CNF-based smart coatings can respond to changes in humidity or temperature, finding applications in self-cleaning surfaces and anti-fogging treatments for glasses and windshields. Similarly, CNF-embedded textiles can adapt to environmental conditions, enhancing comfort and functionality. In biosensors, CNFs integrated with sensing elements enable the detection of biological markers and pollutants, offering solutions for health diagnostics and environmental monitoring. Another promising avenue is the use of CNFs in shape-memory materials and actuators. When combined with CNFs, these materials exhibit improved mechanical strength, flexibility, and reactivity, making them ideal for wearable devices, sensors, and soft robotics. CNF-based actuators, for instance, could provide lightweight, biocompatible alternatives for applications in advanced robotics [101].

Energy-efficient systems also benefit from CNF integration. For instance, thermochromic windows incorporating CNFs adjust transparency based on temperature, reducing building energy consumption [102]. Similarly, CNF-enhanced electrochromic displays and photovoltaic devices promote sustainability in electronic applications [103]. CNFs also contribute to sustainable construction. Laminated wallboard panels using CNFs as binders, combined with additives like fire retardants, offer an eco-friendly alternative to traditional gypsum board. These panels are biodegradable, free of harmful dust, and exhibit improved fire resistance and mechanical properties, making them a safer and more sustainable building material [104]. Table 4 highlights the versatility of CNFs across a range of smart material applications, displaying their capacity to enhance performance, sustainability, and responsiveness. Their integration into advanced systems underscores their potential to drive innovation in environmental sensing, energy efficiency, and adaptive technologies.

## 6. Future Directions and Opportunities

The future of nanotechnology presents exciting opportunities for innovation at increasingly smaller scales. Operating effectively at the nanoscale can revolutionize diverse fields such as medicine, food processing, construction, and beyond. As the applications of cellulose nanomaterials (CNMs) expand, so too will the demand for their production and distribution. However, one significant challenge remains: the high cost of CNMs compared to conventional materials, which continues to limit their widespread adoption [107,108]. Promising advancements are addressing this issue by utilizing agricultural byproducts, such as banana peels, to extract CNFs [109,110]. These innovative approaches leverage the natural abundance and renewability of cellulose, offering a sustainable alternative to synthetic polymers. The material’s unique properties—comparable to those of synthetic counterparts—position CNFs as a viable replacement in many applications.

Recent developments also highlight the use of cellulose for producing rigid nanosized particles, further expanding its potential applications [111]. With continued research and innovation, CNFs could transform industries, enabling capabilities not yet imagined. By reducing production costs and scaling manufacturing, cellulose nanofibers could become a cornerstone material in fields ranging from advanced manufacturing to environmental sustainability. The future of cellulose nanofibers is indeed promising. Their versatility, renewability, and potential to replace synthetic materials underscore their role in driving progress across multiple domains. If fully embraced and optimized, CNFs could unlock transformative possibilities and establish themselves as critical materials for the 21st century.

### 6.1. Emerging Trends in Cellulose Nanomaterials

Cellulose nanomaterials (CNMs) are gaining prominence across various fields, with the medical sector at the forefront of their application. These materials are being utilized for enzyme immobilization, antimicrobial treatments, green catalysis, biosensing, and the synthesis of drug carriers for therapeutic and diagnostic medicine. Their unique properties—such as nanoscale size, hydrophilicity, and biocompatibility—make them ideal drug delivery excipients. CNMs’ large surface area and the negative charge acquired during hydrolysis enable them to bind significant quantities of drugs, offering the potential for precise dosage control [112].

**Figure 4:**
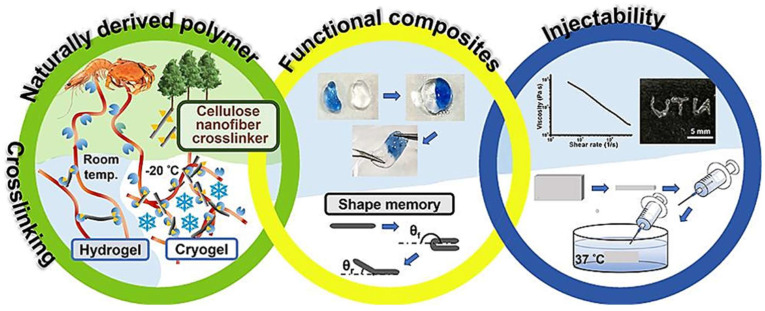
Diagram illustrating the process of using CNFs to create self-healing hydrogel. Reproduced with permission from [113].

As emerging technologies revolutionize medicine, CNMs are poised to catalyze groundbreaking advancements, such as nanoscale devices operating autonomously within the human body. For instance, cellulose nanofibers (CNFs) are used to develop self-healing hydrogels: injectable biomaterials with adjustable, self-repairing properties that hold promise for applications in wound healing and tissue engineering, as shown in Figure 4.

Beyond medicine, CNMs are also driving innovations in electronics. Foldable screens benefit from CNMs’ ability to integrate light-emitting diodes (LEDs) at a nanoscale. These LEDs are stretchable, compressible, and capable of functioning as ultrathin, free-standing films. They can operate on rigid or flexible surfaces and endure mechanical deformation, including curvature radii under 10 µm and tensile strain up to 100% [114].

In energy storage, CNMs have shown potential in lithium–sulfur batteries. A three-dimensional carbonaceous aerogel derived from bacterial cellulose (BC) has been introduced as a flexible framework for sulfur in these batteries. This carbonized BC (CBC) structure offers excellent electrical conductivity and mechanical stability. Its interconnected nanofibrous design prevents the aggregation of sulfur on the cathode surface, enhancing electrochemical performance and sustainability. The synergy of these materials creates more durable and efficient batteries, which are crucial in a modern world reliant on portable devices and renewable energy [115]. Table 5 categorizes the key emerging applications of CNMs and highlights products where they are already making an impact.

### 6.2. Integration with Advanced Manufacturing Technologies

Cellulose, a linear polysaccharide with remarkable potential for advanced material applications, faces significant processing challenges. Unlike many polymers, cellulose degrades before melting and is insoluble in typical organic solvents. Despite these hurdles, advancements in material science have enabled the development of cellulose-based aerogels with exceptional properties, including porosity exceeding 90%, ultralow bulk density (as low as 0.0085 mg/cm^3^), and large specific surface areas. These characteristics make cellulose aerogels ideal for sophisticated applications such as adsorbents and high-performance composites. Nanocellulose-based aerogels are exceptionally versatile due to their ability to integrate various chemical additives, enabling customized materials with specific properties [116]. Recent breakthroughs in overcoming processing challenges include rheology manipulation, 3D Printing CNT/NFC Microfibers, and surface modifications.

Rheological control facilitates the uniform distribution of components like steel fibers in nanocellulose-reinforced composites, enhancing mechanical properties. For example, incorporating CNFs into ultra-high-performance concrete (UHPC) improves tensile strength by 11–23% and transforms the failure mode from single to multiple cracking. This process also reduces fiber settling, ensuring more uniform reinforcement [117].

Advances in 3D printing have produced highly conductive and mechanically robust carbon nanotube (CNT) and nanofibrillated cellulose (NFC) composite microfibers. These materials exhibit a mechanical strength of 247 MPa and electrical conductivity of 216.7 S/cm due to the well-aligned NFC and CNT fibers. The shear-thinning behavior of the CNT-NFC mixture allows precise control during printing, making it suitable for applications in wearable electronics, energy storage, and thermal management [118,119].

Surface modification techniques, such as grafting poly (ε-caprolactone) onto NFC via ring-opening polymerization or polyacrylic acid (PAA) onto cellulose microfibers, enhance cellulose’s functionality. Other methods, including atom transfer radical polymerization of butyl acrylate, create hydrophobic chains on NFC, further broadening its application potential [120].

Bio-based composites are increasingly being developed to meet industrial demands for enhanced mechanical properties and sustainability. However, the potential of cellulose as a material for additive manufacturing remains underexplored, limiting its widespread adoption. Innovations in this area include the following: (1) Cellulose-Based Polymers and Filaments: The development of cellulose-based 3D printing filaments and liquid deposition modeling techniques enables the production of highly sustainable and customizable materials. (2) 4D Printing: Emerging technologies such as 4D printing of cellulose allow the creation of dynamic structures that respond to environmental stimuli, unlocking new possibilities for smart materials. These advancements align with the growing demand for biodegradable, biocompatible materials, showcasing cellulose’s promise as a renewable alternative to petroleum-based resources [121,122]. Integrating advanced manufacturing technologies with nanocellulose aerogels highlights their potential to revolutionize various industries. The ability to tailor these materials for specific applications, combined with innovative processing techniques, positions cellulose nanomaterials as a cornerstone for producing high-value, sustainable, and performance-enhanced products.

### 6.3. Sustainability and Life Cycle Considerations

The sustainability potential of cellulose nanofiber (CNF) composites has garnered significant attention due to their biodegradability, renewability, and minimal environmental impact. Derived from natural resources, primarily plant biomass, CNFs offer an eco-friendly alternative to synthetic fibers. Research demonstrates that CNFs degrade much faster than petroleum-based polymers, reducing landfill waste and mitigating the environmental harm caused by non-biodegradable materials [123]. A key factor in the sustainability of CNF-based products is the environmental footprint of their production processes. Traditional cellulose extraction methods, such as chemical pulping, often involve intensive use of chemicals, water, and energy, leading to waste and pollution. However, advancements in sustainable production methods are transforming this landscape. Emerging techniques emphasize mechanical fibrillation and enzymatic treatments, which minimize hazardous byproducts and reduce the reliance on harsh chemicals. These approaches align with global sustainability goals, emphasizing greener manufacturing practices in the materials industry [124]. Mechanical fibrillation, for instance, can significantly lower energy consumption compared to traditional methods. Enzymatic treatments further enhance eco-friendliness by replacing chemical-intensive processes with biodegradable alternatives [125].

The sustainability of CNF composites also depends on their integration throughout the material life cycle, particularly the choice of the matrix material. When CNFs are combined with biodegradable polymers like starch or polylactic acid (PLA), the resulting composites maintain their positive environmental impact. These materials degrade more completely, aligning with sustainability objectives. In contrast, pairing CNFs with non-biodegradable matrices limits their environmental benefits, as these composites do not fully decompose. Careful selection of compatible, biodegradable matrices is essential to achieving life cycle sustainability. CNF composites are lightweight, which reduces energy demands during transportation and handling. This characteristic contributes to lower greenhouse gas emissions over the product’s lifecycle [126].

End-of-life strategies play a critical role in the sustainability of CNF-based products. CNF composites offer promising opportunities for composting and recycling, depending on the matrix and modification techniques used. Unlike traditional synthetic materials, CNFs can often be composted, significantly reducing landfill waste. Recycling CNF composites supports a circular economy by extending material life, reducing the need for virgin resources, and enabling the creation of new products from recycled materials [127].

CNFs also present significant environmental advantages as a carbon sink. Because cellulose originates from plants that absorb carbon during growth, CNF-based products can sequester carbon when used in long-term applications such as construction materials or furniture. Utilizing plant-derived fibers supports the transition to a bio-based economy, reducing reliance on fossil fuels and promoting renewable resource use [128]. Incorporating CNFs into industrial applications can play a meaningful role in combating climate change by leveraging their natural carbon storage capabilities.

While the adoption of CNFs marks a significant step toward greener materials, challenges remain, particularly in scaling environmentally friendly production techniques. Overcoming these barriers will require continued innovation in manufacturing processes and matrix compatibility. Nevertheless, CNFs hold immense promise for advancing sustainable materials across diverse industries, paving the way for a more eco-friendly future.

### 6.4. Prospects for Commercialization and Market Growth

**Figure 5:**
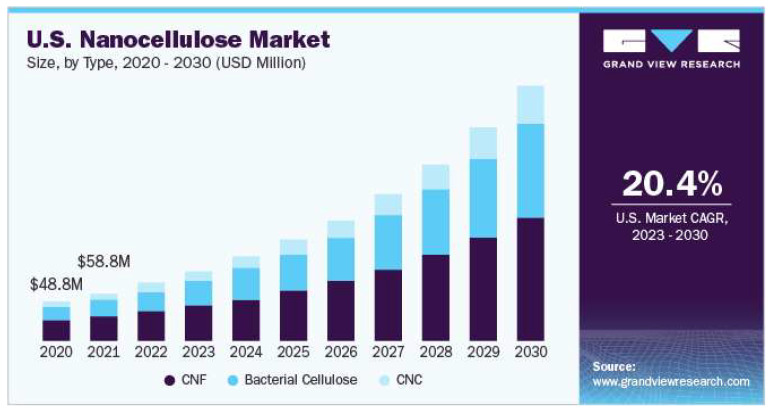
U.S. nanocellulose market. Copyright © 2025 Grand View Research, Inc. [129].

The commercialization of CNFs is progressing rapidly, fueled by their remarkable properties, including high mechanical strength, lightweight structure, biodegradability, and excellent thermal stability. As renewable materials, CNFs are gaining traction across a wide range of industries, from sustainable packaging and biomedical engineering to construction materials and electronic devices. Their ability to replace petroleum-based materials positions CNFs as a critical driver in the global transition toward greener, more sustainable technologies [129]. Figure 5 showcases the current trends of the nanocellulose market, consisting of CNFs, bacterial cellulose, and CNCs.

The global market for CNFs is anticipated to grow significantly, with a projected compound annual growth rate (CAGR) exceeding 19% from 2023 to 2033. By 2031, the market is expected to surpass USD 7 billion, driven by increasing demand for eco-friendly solutions in packaging and lightweight composites. Regions such as North America, Europe, and East Asia are leading this growth, propelled by robust innovation, production capabilities, and stringent environmental regulations. Industry leaders like Nippon Paper, Kruger Inc., Borregaard, and Sappi are pioneering advancements in CNF production, fostering growth across multiple sectors [130,131].

Recent technological advancements in CNF production are pivotal in reducing costs and enhancing scalability. Techniques such as enzymatic pretreatment and TEMPO oxidation have improved energy efficiency and environmental performance, making CNFs more accessible for high-value applications. CNFs are replacing single-use plastics in food packaging, offering an eco-friendly alternative that meets consumer and regulatory demands on biodegradable packaging. CNFs’ biocompatibility and ability to mimic natural extracellular matrices have made them ideal for wound healing, tissue engineering, and drug delivery systems in biomedical applications. In the electronics industry, transparent conductive films, flexible displays, and sensors are emerging as key applications for CNFs, showcasing their versatility in cutting-edge technologies [132,133].

Despite their promise, the widespread adoption of CNFs faces several challenges: (1) High Initial Investments: Scaling up production facilities requires significant capital expenditure. (2) Energy-Intensive Production: While recent innovations have reduced energy requirements, further optimization is necessary to enhance cost-effectiveness. (3) Lack of Standardized Regulations: The absence of uniform standards for CNF production and application hampers market development. Addressing these obstacles will require concerted efforts through collaboration among industry, academia, and government. Public–private partnerships and increased investment in research and development will be crucial to overcoming these barriers and unlocking the full potential of CNFs [134].

Regional dynamics play a crucial role in shaping the commercialization and adoption of CNFs. The European region leads the market, driven by stringent regulations against single-use plastics and a well-established pulp and paper industry. The Asia–Pacific region’s rapid industrialization and demand for lightweight composites in the automotive and construction sectors is spurring growth. Japan and South Korea are leveraging CNFs in advanced materials for aerospace and automotive applications. South America and Africa are beginning to explore CNFs’ potential as sustainable alternatives to conventional materials, presenting opportunities for market expansion [107,135].

Looking ahead, CNFs are poised to become integral to the development of a circular economy. As industries and governments increasingly invest in sustainable materials, the demand for CNFs will surge, creating economic opportunities while addressing pressing environmental challenges. The growing adoption of CNFs across sectors is expected to drive innovation, enhance sustainability, and reduce environmental footprints. As the market expands, CNFs are set to become a cornerstone of the green transition, supporting global efforts to combat climate change and foster sustainable development. For further exploration of these trends, market reports from Persistence Market Research, Grand View Research, and Future Market Insights, along with specialized scientific journals and technological forecasting studies, provide valuable insights [136].

## 7. Conclusions

Cellulose nanofibers (CNFs), nanomaterials (CNMs), and cellulose nanocrystals (CNCs) represent a paradigm shift in sustainable material science and manufacturing engineering. Derived from renewable sources such as wood, cotton, and agricultural byproducts, these materials exemplify versatility, functionality, and environmental stewardship. This review has provided a comprehensive overview of their extraction methods, structural characteristics, functional properties, and diverse applications. Advanced extraction and processing techniques—including chemical, mechanical, and enzymatic methods—enable the development of materials with tailored properties for specialized applications. While challenges like scalability, cost efficiency, and uniformity persist, innovative approaches such as electrospinning and melt spinning offer promising solutions, paving the way for broader adoption.

The remarkable structural and functional properties of cellulose-based nanomaterials, including high strength, durability, thermal stability, and optical transparency, position them as transformative alternatives to conventional synthetic materials. These attributes have propelled their use across diverse industries, ranging from sustainable packaging and insulation to renewable energy and smart materials. Their inherent biocompatibility and environmental sustainability align with global efforts to reduce ecological footprints, making cellulose-based nanomaterials indispensable in the pursuit of sustainable development. As advancements in eco-friendly production methods and hybrid composites continue, CNFs, CNMs, and CNCs are becoming increasingly viable for large-scale industrial applications.

A key strength of cellulose-based nanomaterials lies in their ability to enhance nanocomposite technologies. Leveraging mechanisms such as hydrogen bonding and mechanical interlocking, CNFs significantly improve the mechanical and functional properties of composites. Modern fabrication techniques, such as 3D printing and solution casting, enable precise customization, facilitating applications in high-impact industries like automotive, biomedical, and electronics. However, while these materials offer numerous advantages, several challenges persist. The difficulty in achieving homogeneous dispersion within composite matrices, optimizing large-scale production processes, and improving long-term stability remains a significant hurdle. Additionally, the influence of processing parameters on the final properties of nanocellulose composites needs further investigation to ensure consistency in performance across applications.

Future research should prioritize overcoming these challenges by enhancing large-scale production techniques, establishing cost-effective functionalization methods, and optimizing nanocellulose–polymer interactions to improve composite performance. Tackling these issues will enable cellulose-based nanomaterials to play a crucial role in the advancement of next-generation, high-performance, and sustainable materials, supporting global initiatives for environmental conservation and resource efficiency.

## Figures and Tables

**Table 1:** Techniques for cellulose nanofibers: cotton vs. wood pulp [24,25,26].

Cellulose Source	Processing Techniques	Key Details
Cotton	Chemical process	Acidified sodium chlorite solution removes lignin. Potassium hydroxide solution eliminates hemicelluloses. Hydrochloric acid treatment followed by distilled water washing after each step.
	Mechanical process	Cotton fiber bundles are ground at 1800 rpm, refining fibers into CNFs.
Wood pulp	Mechanical process	Wood microparticles are ground, screened, and immersed in distilled water for 5 h. Processed at two wt. % concentration and passed through a disk grinder 6 times.
	Characteristics	High cellulose purity and strong bonding. Excellent intrinsic physical properties, biodegradability, and no axial thermal expansion.
	Processing details	Demonstrated on Paulownia wood: particles ground to 420 μm, with the rotor speed reduced stepwise from 2800 to 1800 rpm, producing fine CNFs.
	Eco-friendly Mechanical processing	Wood-based CNFs, requiring no chemical treatments, offer an advantage regarding limited environmental impact.

**Table 2:** Potential applications of CNFs and their key properties.

Field	Key Properties of CNFs	Applications
Packaging	High mechanical strength, biodegradability, and low density.	Eco-friendly packaging materials, biodegradable films, and coatings for food packaging.
Composites	High strength-to-weight ratio, reinforcing capability, and flexibility.	Reinforcement in composite materials for automotive, aerospace, and construction to reduce weight.
Biomedical	Biocompatibility, biodegradability, non-toxicity, and lightweight.	Wound dressings, drug delivery systems, tissue engineering scaffolds, and medical implants.
Paper and pulp industry	High surface area, mechanical strength, and fibrillation ability.	Strength enhancement in paper, improved paper coatings, and enhanced functionality of paper products.
Electronics	High surface area, conductivity (when modified), and flexibility.	Flexible electronics, sensors, supercapacitors, and energy storage devices.
Environmental applications	Biodegradability, non-toxicity, and adsorption capability.	Water filtration, air purification, and adsorption of oil spills and heavy metals.
Textiles	High strength, flexibility, lightweight, and antimicrobial properties.	High-performance fabrics, wearable textiles, and antimicrobial coatings for clothing.
Energy storage	High surface area, conductivity (when modified), and lightweight.	Batteries, supercapacitors, and fuel cells to enhance energy efficiency and storage capacity.
Coatings and films	Transparency, barrier properties, high surface area, and flexibility.	Protective coatings, transparent films for electronics, and food packaging films to improve product shelf life.
Construction	High strength-to-weight ratio, fire resistance, and sustainability	Construction materials such as lightweight concrete, insulation materials, and sustainable composites.

**Table 3:** The categorization and applications of CNCs and CNMs in manufacturing. Modified from [88].

High-Volume Applications	Low-Volume Applications	Emerging Applications
Automotive body	Aerospace structures	Purification technologies
Automotive interior	Aerospace interiors	Flexible electronics
Packaging coatings	Wallboard facing	Recyclable electronics
Paper coatings	Insulation materials	Biomedical sensors
Packaging filler		
Replacement for plastic packaging		
Replacement for plastic film		

**Table 4:** Applications of cellulose nanofibers in smart materials and responsive systems.

Application	Functionality	Examples	Reference
Smart coatings	Respond to stimuli like humidity and temperature for enhanced functionality.	Self-cleaning surfaces and anti-fogging treatments.	[101,105]
Responsive textiles	Adapt to environmental changes for improved comfort and functionality.	Clothing that adjusts to humidity and temperature.	[105]
Biosensors	Detect biological markers or pollutants for health and environmental monitoring.	Health diagnostics and pollutant detection devices.	[106]
Shape-memory materials	React to stimuli with improved mechanical properties and reactivity.	Wearable devices, soft robotics, and flexible sensors.	[106]
Energy-efficient Systems	Modify transparency or conductivity for energy savings.	Thermochromic windows and electrochromic displays.	[106]

**Table 5:** Various emerging trends for cellulose nanomaterials.

Medical Applications	Electronics	Batteries
Drug delivery systems Wound healing Tissue engineering Antimicrobial treatment	Foldable smartphones Flexible displays Pacemakers Heads-up displays (HUDs)	Easily recyclable materials Energy-efficient automotive applications Enhanced performance with metal composites

## References

[1] Holtzapple M.T., Caballero B. (2003). Cellulose. Encyclopedia of Food Sciences and Nutrition.

[2] Thomas B., Raj M.C., Joy J., Moores A., Drisko G.L., Sanchez C. (2018). Nanocellulose, a Versatile Green Platform: From Biosources to Materials and Their Applications. Chem. Rev..

[3] Liu X., Jiang Y., Wang L., Song X., Qin C., Wang S. (2020). Tuning of Size and Properties of Cellulose Nanofibers Isolated from Sugarcane Bagasse by Endoglucanase-Assisted Mechanical Grinding. Ind. Crops Prod..

[4] Mishra R.K., Ha S.K., Verma K., Tiwari S.K. (2018). Recent Progress in Selected Bio-Nanomaterials and Their Engineering Applications: An Overview. J. Sci. Adv. Mater. Devices.

[5] Barari B., Omrani E., Moghadam A.D., Menezes P.L., Pillai K.M., Rohatgi P.K. (2016). Mechanical, Physical and Tribological Characterization of Nano-Cellulose Fibers Reinforced Bio-Epoxy Composites: An Attempt to Fabricate and Scale the ‘Green’ Composite. Carbohydr. Polym..

[6] Kassie B.B., Daget T.M., Tassew D.F. (2024). Synthesis, Functionalization, and Commercial Application of Cellulose-Based Nanomaterials. Int. J. Biol. Macromol..

[7] Kumar R., Kumar G., Bhat R., Kumar A., Nguyen T.A., Sharma S. (2022). Chapter 18—Nanocellulose: Fascinating and Sustainable Nanomaterial for Papermaking. Nanotechnology in Paper and Wood Engineering.

[8] Norizan M.N., Shazleen S.S., Alias A.H., Sabaruddin F.A., Asyraf M.R.M., Zainudin E.S., Abdullah N., Samsudin M.S., Kamarudin S.H., Norrrahim M.N.F. (2022). Nanocellulose-Based Nanocomposites for Sustainable Applications: A Review. Nanomaterials.

[9] Shazleen S.S., Sabaruddin F.A., Ando Y., Ariffin H. (2023). Optimization of Cellulose Nanofiber Loading and Processing Conditions during Melt Extrusion of Poly(3-Hydroxybutyrate-Co-3-Hydroxyhexanoate) Bionanocomposites. Polymers.

[10] Quan G., Wu Y., Li W., Li D., Liu X., Wang K., Dai S., Xiao L., Ao Y. (2024). Construction of Cellulose Nanofiber/Carbon Nanotube Synergistic Network on Carbon Fiber Surface to Enhance Mechanical Properties and Thermal Conductivity of Composites. Compos. Sci. Technol..

[11] Jung H., Shin G., Kwak H., Hao L.T., Jegal J., Kim H.J., Jeon H., Park J., Oh D.X. (2023). Review of Polymer Technologies for Improving the Recycling and Upcycling Efficiency of Plastic Waste. Chemosphere.

[12] Phanthong P., Reubroycharoen P., Hao X., Xu G., Abudula A., Guan G. (2018). Nanocellulose: Extraction and Application. Carbon Resour. Convers..

[13] Dufresne A. (2013). Nanocellulose: A New Ageless Bionanomaterial. Mater. Today.

[14] Koh J., Clark M. (2011). 3—Dyeing of Cellulosic Fibres. Handbook of Textile and Industrial Dyeing.

[15] Felgueiras C., Azoia N.G., Gonçalves C., Gama M., Dourado F. (2021). Trends on the Cellulose-Based Textiles: Raw Materials and Technologies. Front. Bioeng. Biotechnol..

[16] Channab B.-E., Idrissi A.E., Essamlali Y., Zahouily M. (2024). Nanocellulose: Structure, Modification, Biodegradation and Applications in Agriculture as Slow/Controlled Release Fertilizer, Superabsorbent, and Crop Protection: A Review. J. Environ. Manag..

[17] Verma C., Chhajed M., Gupta P., Roy S., Maji P.K. (2021). Isolation of Cellulose Nanocrystals from Different Waste Bio-Mass Collating Their Liquid Crystal Ordering with Morphological Exploration. Int. J. Biol. Macromol..

[18] Dufresne A. (2020). Preparation and Properties of Cellulose Nanomaterials. Pap. Biomater..

[19] Ren H., Huang Y., Yang W., Ling Z., Liu S., Zheng S., Li S., Wang Y., Pan L., Fan W. (2024). Emerging Nanocellulose from Agricultural Waste: Recent Advances in Preparation and Applications in Biobased Food Packaging. Int. J. Biol. Macromol..

[20] Chaka K.T. (2022). Extraction of Cellulose Nanocrystals from Agricultural By-Products: A Review. Green Chem. Lett. Rev..

[21] Singh S.K., Dhepe P.L. (2022). Alpha-, Beta- and Gamma-Cellulose Quantification and Two-Stage Concentrated-Dilute Acid Lignin Recovery from Three Rice Husks: Lignin Characterization and Depolymerization. Waste Biomass Valorization.

[22] Nishiyama Y., Sugiyama J., Chanzy H., Langan P. (2003). Crystal Structure and Hydrogen Bonding System in Cellulose Iα from Synchrotron X-Ray and Neutron Fiber Diffraction. J. Am. Chem. Soc..

[23] Dri F.L., Shang S., Hector L.G., Saxe P., Liu Z.-K., Moon R.J., Zavattieri P.D. (2014). Anisotropy and Temperature Dependence of Structural, Thermodynamic, and Elastic Properties of Crystalline Cellulose I*_β_*: A First-Principles Investigation. Model. Simul. Mater. Sci. Eng..

[24] Li J., Song Z., Li D., Shang S., Guo Y. (2014). Cotton Cellulose Nanofiber-Reinforced High Density Polyethylene Composites Prepared with Two Different Pretreatment Methods. Ind. Crops Prod..

[25] Prakash Menon M., Selvakumar R., Suresh Kumar P., Ramakrishna S. (2017). Extraction and Modification of Cellulose Nanofibers Derived from Biomass for Environmental Application. RSC Adv..

[26] Yousefi H., Azari V., Khazaeian A. (2018). Direct Mechanical Production of Wood Nanofibers from Raw Wood Microparticles with No Chemical Treatment. Ind. Crops Prod..

[27] Yi T., Zhao H., Mo Q., Pan D., Liu Y., Huang L., Xu H., Hu B., Song H. (2020). From Cellulose to Cellulose Nanofibrils—A Comprehensive Review of the Preparation and Modification of Cellulose Nanofibrils. Materials.

[28] Ghasemlou M., Daver F., Ivanova E.P., Habibi Y., Adhikari B. (2021). Surface Modifications of Nanocellulose: From Synthesis to High-Performance Nanocomposites. Prog. Polym. Sci..

[29] Valipouri A. (2017). Production Scale Up of Nanofibers: A Review. J. Text. Polym..

[30] Maduna L., Patnaik A. (2024). Challenges Associated with the Production of Nanofibers. Processes.

[31] Huang Y., Song J., Yang C., Long Y., Wu H. (2019). Scalable Manufacturing and Applications of Nanofibers. Mater. Today.

[32] Patnaik A., Anandjiwala R.D. (2016). An Optimized Melt Spinning Process to Increase the Productivity of Nanofiber Materials. J. Ind. Text..

[33] Altaner C.M., Thomas L.H., Fernandes A.N., Jarvis M.C. (2014). How Cellulose Stretches: Synergism between Covalent and Hydrogen Bonding. Biomacromolecules.

[34] Tanpichai S., Boonmahitthisud A., Soykeabkaew N., Ongthip L. (2022). Review of the Recent Developments in All-Cellulose Nanocomposites: Properties and Applications. Carbohydr. Polym..

[35] Qi X., Qiao J., Liu M., Zhang Y., Ma Q., Guo X., Wu Y. (2025). Multifunctional Cellulose Nanofibrils-Based Composite Film with Superior Mechanical Strength and Flame Retardancy. Int. J. Biol. Macromol..

[36] Mokhena T.C., Sadiku E.R., Mochane M.J., Ray S.S., John M.J., Mtibe A. (2021). Mechanical Properties of Cellulose Nanofibril Papers and Their Bionanocomposites: A Review. Carbohydr. Polym..

[37] Gan P.G., Sam S.T., bin Abdullah M.F., Omar M.F. (2020). Thermal Properties of Nanocellulose-Reinforced Composites: A Review. J. Appl. Polym. Sci..

[38] Gumowska A., Kowaluk G., Labidi J., Robles E. (2019). Barrier Properties of Cellulose Nanofiber Film as an External Layer of Particleboard. Clean Technol. Environ. Policy.

[39] Zhang Y., Hao N., Lin X., Nie S. (2020). Emerging Challenges in the Thermal Management of Cellulose Nanofibril-Based Supercapacitors, Lithium-Ion Batteries and Solar Cells: A Review. Carbohydr. Polym..

[40] Oesef K., Cranston E.D., Abdin Y. (2024). Current Advances in Processing and Modification of Cellulose Nanofibrils for High-Performance Composite Applications. Mater. Des..

[41] D’Acierno F., Michal C.A., MacLachlan M.J. (2023). Thermal Stability of Cellulose Nanomaterials. Chem. Rev..

[42] Khatri M., Ahmed F., Shaikh I., Phan D.-N., Khan Q., Khatri Z., Lee H., Kim I.S. (2017). Dyeing and Characterization of Regenerated Cellulose Nanofibers with Vat Dyes. Carbohydr. Polym..

[43] Khatri M., Ahmed F.E., Al-Juboori R.A., Khanzada N.K., Hilal N. (2024). Reusable Environmentally Friendly Electrospun Cellulose Acetate/Cellulose Nanocrystals Nanofibers for Methylene Blue Removal. J. Environ. Chem. Eng..

[44] Zhang Y., Liu Y., Dong C., Li R., Zhang X., Wang T., Zhang K. (2024). Transparent, Thermal Stable, Water Resistant and High Gas Barrier Films from Cellulose Nanocrystals Prepared by Reactive Deep Eutectic Solvents. Int. J. Biol. Macromol..

[45] Hussain M., Khan S.M., Shafiq M., Abbas N. (2024). A Review on PLA-Based Biodegradable Materials for Biomedical Applications. Giant.

[46] Ali M.S., Bhunia P., Samanta A.P., Orasugh J.T., Chattopadhyay D. (2022). Transdermal Therapeutic System: Study of Cellulose Nanocrystals Influenced Methylcellulose-Chitosan Bionanocomposites. Int. J. Biol. Macromol..

[47] Kim J., Muthoka R.M., Kim H.C., Panicker P.S., Yoon H., Kim J. (2021). Mechanical, Electrical and Optical Properties of Cellulose Nanofiber Films Modified Using Polydopamine Coating. Proceedings of the Nano-, Bio-, Info-Tech Sensors and Wearable Systems.

[48] Li R., Tian D., Chen L., Zhuang B., Feng H., Li Q., Yu L., Ling Y. (2023). The Application of Cellulose Nanofibrils in Energy Systems. Batteries.

[49] Kang C.S., Kim J.K., Lee C.-S., Chang H., Cho Y.H., Prasad C., Choi H.Y. (2024). Facile Fabrication and Characterization of MXene/Cellulose Composites for Electrical Properties, Electric Heating Performance. Fash. Text..

[50] Poisson J., Zhang K. (2024). Unique Optical Properties of Cellulosic Materials. Acc. Mater. Res..

[51] Jaiswal A.K., Hokkanen A., Kumar V., Mäkelä T., Harlin A., Orelma H. (2021). Thermoresponsive Nanocellulose Films as an Optical Modulation Device: Proof-of-Concept. ACS Appl. Mater. Interfaces.

[52] Niskanen I., Zhang K., Karzarjeddi M., Liimatainen H., Shibata S., Hagen N., Heikkilä R., Yoda H., Otani Y. (2022). Optical Properties of Cellulose Nanofibre Films at High Temperatures. J. Polym. Res..

[53] Tanpichai S., Biswas S.K., Witayakran S., Yano H. (2020). Optically Transparent Tough Nanocomposites with a Hierarchical Structure of Cellulose Nanofiber Networks Prepared by the Pickering Emulsion Method. Compos. Part Appl. Sci. Manuf..

[54] Klemm D., Kramer F., Moritz S., Lindström T., Ankerfors M., Gray D., Dorris A. (2011). Nanocelluloses: A New Family of Nature-Based Materials. Angew. Chem. Int. Ed..

[55] Ferreira F.V., Souza A.G., Ajdary R., de Souza L.P., Lopes J.H., Correa D.S., Siqueira G., Barud H.S., Rosa D.d.S., Mattoso L.H.C. (2023). Nanocellulose-Based Porous Materials: Regulation and Pathway to Commercialization in Regenerative Medicine. Bioact. Mater..

[56] Lin N., Dufresne A. (2014). Nanocellulose in Biomedicine: Current Status and Future Prospect. Eur. Polym. J..

[57] Hubbe M., Rojas O., Lucia L., Sain M. (2008). Cellulosic Nanocomposites: A Review. BioResources.

[58] Guadagno L., Vertuccio L., Barra G., Naddeo C., Sorrentino A., Lavorgna M., Raimondo M., Calabrese E. (2021). Eco-Friendly Polymer Nanocomposites Designed for Self-Healing Applications. Polymer.

[59] Kamdem Tamo A., Doench I., Morales Helguera A., Hoenders D., Walther A., Madrazo A.O. (2020). Biodegradation of Crystalline Cellulose Nanofibers by Means of Enzyme Immobilized-Alginate Beads and Microparticles. Polymers.

[60] Xi P., Quan F., Sun Y., Jiang Y. (2022). Cellulose Nanofibers Reinforced Nanocomposites with High Strength and Toughness by Tunable Wet-Drawing and Ionic Cross-Linking Method. Compos. Part B Eng..

[61] Trivedi A.K., Gupta M.K., Singh H. (2023). PLA Based Biocomposites for Sustainable Products: A Review. Adv. Ind. Eng. Polym. Res..

[62] Ebrahimi F., Dana H.R. (2022). Poly Lactic Acid (PLA) Polymers: From Properties to Biomedical Applications. Int. J. Polym. Mater. Polym. Biomater..

[63] Trivedi A.K., Gupta M.K. (2024). PLA Based Biodegradable Bionanocomposite Filaments Reinforced with Nanocellulose: Development and Analysis of Properties. Sci. Rep..

[64] Shuai C., Yuan X., Yang W., Peng S., He C., Feng P., Qi F., Wang G. (2020). Cellulose Nanocrystals as Biobased Nucleation Agents in Poly-l-Lactide Scaffold: Crystallization Behavior and Mechanical Properties. Polym. Test..

[65] Kargarzadeh H., Huang J., Lin N., Ahmad I., Mariano M., Dufresne A., Thomas S., Gałęski A. (2018). Recent Developments in Nanocellulose-Based Biodegradable Polymers, Thermoplastic Polymers, and Porous Nanocomposites. Prog. Polym. Sci..

[66] Samyn P., Cosemans P., Chandroth A.M., Takahashi K., Everaerts J. (2025). Mechanical Properties of Spray-Coated Nanocellulose Coatings. Surf. Coat. Technol..

[67] Henderson D., Zhang X., Mao Y., Hu L., Briber R.M., Wang H. (2021). Cellulose Nanocomposites of Cellulose Nanofibers and Molecular Coils. J. Compos. Sci..

[68] Neagu R.C., Cuénoud M., Berthold F., Bourban P.-E., Gamstedt E.K., Lindström M., Månson J.-A.E. (2012). The Potential of Wood Fibers as Reinforcement in Cellular Biopolymers. J. Cell. Plast..

[69] Baghaei B., Skrifvars M. (2020). All-Cellulose Composites: A Review of Recent Studies on Structure, Properties and Applications. Molecules.

[70] Moon R.J., Martini A., Nairn J., Simonsen J., Youngblood J. (2011). Cellulose Nanomaterials Review: Structure, Properties and Nanocomposites. Chem. Soc. Rev..

[71] Gray D. (2013). Nanocellulose: From Nature to High Performance Tailored Material. Holzforschung.

[72] Nagarajan K.J., Ramanujam N.R., Sanjay M.R., Siengchin S., Rajan B.S., Basha K.S., Madhu P., Raghav G.R. (2021). A Comprehensive Review on Cellulose Nanocrystals and Cellulose Nanofibers: Pretreatment, Preparation, and Characterization. Polym. Compos..

[73] Lee K.-Y., Aitomäki Y., Berglund L.A., Oksman K., Bismarck A. (2014). On the Use of Nanocellulose as Reinforcement in Polymer Matrix Composites. Compos. Sci. Technol..

[74] Siró I., Plackett D. (2010). Microfibrillated Cellulose and New Nanocomposite Materials: A Review. Cellulose.

[75] Anusiya G., Jaiganesh R. (2022). A Review on Fabrication Methods of Nanofibers and a Special Focus on Application of Cellulose Nanofibers. Carbohydr. Polym. Technol. Appl..

[76] Huczko A. (2000). Template-Based Synthesis of Nanomaterials. Appl. Phys. A.

[77] Joseph B., Sagarika V.K., Sabu C., Kalarikkal N., Thomas S. (2020). Cellulose Nanocomposites: Fabrication and Biomedical Applications. J. Bioresour. Bioprod..

[78] Latif M., Jiang Y., Kim J. (2023). 3D Printing of Concentrated Nanocellulose Material: The Critical Role of Substrates on the Shape Fidelity and Mechanical Properties. Carbohydr. Polym..

[79] Marras S.I., Zuburtikudis I., Panayiotou C. (2010). Solution Casting versus Melt Compounding: Effect of Fabrication Route on the Structure and Thermal Behavior of Poly(l-Lactic Acid) Clay Nanocomposites. J. Mater. Sci..

[80] Sato A., Kabusaki D., Okumura H., Nakatani T., Nakatsubo F., Yano H. (2016). Surface Modification of Cellulose Nanofibers with Alkenyl Succinic Anhydride for High-Density Polyethylene Reinforcement. Compos. Part Appl. Sci. Manuf..

[81] Soofi M., Alizadeh A., Hamishehkar H., Almasi H., Roufegarinejad L. (2021). Preparation of Nanobiocomposite Film Based on Lemon Waste Containing Cellulose Nanofiber and Savory Essential Oil: A New Biodegradable Active Packaging System. Int. J. Biol. Macromol..

[82] Chu Y., Sun Y., Wu W., Xiao H. (2020). Dispersion Properties of Nanocellulose: A Review. Carbohydr. Polym..

[83] Wang L., Okada K., Sodenaga M., Hikima Y., Ohshima M., Sekiguchi T., Yano H. (2018). Effect of Surface Modification on the Dispersion, Rheological Behavior, Crystallization Kinetics, and Foaming Ability of Polypropylene/Cellulose Nanofiber Nanocomposites. Compos. Sci. Technol..

[84] Omran A.A.B., Mohammed A.A.B.A., Sapuan S.M., Ilyas R.A., Asyraf M.R.M., Rahimian Koloor S.S., Petrů M. (2021). Micro- and Nanocellulose in Polymer Composite Materials: A Review. Polymers.

[85] O’Banion E.E., Es-haghi S.S. (2023). Rheological Behavior of Cellulose Nanofibril Suspensions with Varied Levels of Fines and Solid Content. Polymer.

[86] Arvidsson R., Nguyen D., Svanström M. (2015). Life Cycle Assessment of Cellulose Nanofibrils Production by Mechanical Treatment and Two Different Pretreatment Processes. Environ. Sci. Technol..

[87] Gallo Stampino P., Riva L., Punta C., Elegir G., Bussini D., Dotelli G. (2021). Comparative Life Cycle Assessment of Cellulose Nanofibres Production Routes from Virgin and Recycled Raw Materials. Molecules.

[88] Market Projections of Cellulose Nanomaterial-Enabled Products—Part 1: Applications | US Forest Service Research and Development. https://research.fs.usda.gov/treesearch/46174.

[89] Djafari Petroudy S.R., Shojaeiarani J., Chabot B. (2023). Recent Advances in Isolation, Characterization, and Potential Applications of Nanocellulose-Based Composites: A Comprehensive Review. J. Nat. Fibers.

[90] Hossain R., Tajvidi M., Bousfield D., Gardner D.J. (2022). Recyclable Grease-Proof Cellulose Nanocomposites with Enhanced Water Resistance for Food Serving Applications. Cellulose.

[91] Liu W., Liu K., Du H., Zheng T., Zhang N., Xu T., Pang B., Zhang X., Si C., Zhang K. (2022). Cellulose Nanopaper: Fabrication, Functionalization, and Applications. Nano-Micro Lett..

[92] Nurazzi N.M., Jenol M.A., Kamarudin S.H., Aisyah H.A., Hao L.C., Yusuff S.M., Amira M.R.N., Harussani M.M., Norrrahim M.N.F., Ilyas R.A., Sapuan S.M., Norrrahim M.N.F., Ilyas R.A., Soutis C. (2022). 19—Nanocellulose Composites in the Automotive Industry. Industrial Applications of Nanocellulose and Its Nanocomposites.

[93] Ashraf R., Sofi H.S., Akram T., Rather H.A., Abdal-hay A., Shabir N., Vasita R., Alrokayan S.H., Khan H.A., Sheikh F.A. (2020). Fabrication of Multifunctional Cellulose/TiO_2_/Ag Composite Nanofibers Scaffold with Antibacterial and Bioactivity Properties for Future Tissue Engineering Applications. J. Biomed. Mater. Res. A.

[94] Sharip N.S., Ariffin H. (2019). Cellulose Nanofibrils for Biomaterial Applications. Mater. Today Proc..

[95] Guo X., Gao H., Zhang J., Zhang L., Shi X., Du Y. (2021). One-Step Electrochemically Induced Counterion Exchange to Construct Free-Standing Carboxylated Cellulose Nanofiber/Metal Composite Hydrogels. Carbohydr. Polym..

[96] Rashki S., Shakour N., Yousefi Z., Rezaei M., Homayoonfal M., Khabazian E., Atyabi F., Aslanbeigi F., Safaei Lapavandani R., Mazaheri S. (2021). Cellulose-Based Nanofibril Composite Materials as a New Approach to Fight Bacterial Infections. Front. Bioeng. Biotechnol..

[97] Zanette R.D.S.S., Fayer L., Vasconcellos R., De Oliveira L.F.C., Maranduba C.M.D.C., De Alvarenga É.L.F.C., Martins M.A., Brandão H.D.M., Munk M. (2023). Cytocompatible and Osteoinductive Cotton Cellulose Nanofiber/Chitosan Nanobiocomposite Scaffold for Bone Tissue Engineering. Biomed. Mater..

[98] Wasim M., Shi F., Liu J., Khan M.R., Farooq A., Sanbhal N., Alfred M., Xin L., Yajun C., Zhao X. (2021). Extraction of Cellulose to Progress in Cellulosic Nanocomposites for Their Potential Applications in Supercapacitors and Energy Storage Devices. J. Mater. Sci..

[99] Illa M.P., Pathak A.D., Sharma C.S., Khandelwal M. (2020). Bacterial Cellulose–Polyaniline Composite Derived Hierarchical Nitrogen-Doped Porous Carbon Nanofibers as Anode for High-Rate Lithium-Ion Batteries. ACS Appl. Energy Mater..

[100] Choudhury R.R., Sahoo S.K., Gohil J.M. (2020). Potential of Bioinspired Cellulose Nanomaterials and Nanocomposite Membranes Thereof for Water Treatment and Fuel Cell Applications. Cellulose.

[101] Sharma A., Thakur M., Bhattacharya M., Mandal T., Goswami S. (2019). Commercial Application of Cellulose Nano-Composites—A Review. Biotechnol. Rep..

[102] Feng K., Lu M., Wei G., Tang F., Jin Z. (2022). Cellulose Nanocrystals/Poly(3,4-Ethylenedioxythiophene) Photonic Crystal Composites with Electrochromic Properties for Smart Windows, Displays, and Anticounterfeiting/Encryption Applications. ACS Appl. Nano Mater..

[103] Lang A.W., Österholm A.M., Reynolds J.R. (2019). Paper-Based Electrochromic Devices Enabled by Nanocellulose-Coated Substrates. Adv. Funct. Mater..

[104] Hafez I., Tajvidi M. (2020). Laminated Wallboard Panels Made with Cellulose Nanofibrils as a Binder: Production and Properties. Materials.

[105] Yu R., Prabhakar M.N., Feng J., Yang Y., Hong S.H., Song J. (2025). Enhancing the Mechanical Properties of Flax Fiber-Reinforced Epoxy Composites through Cellulose Nanofiber Incorporation. Ind. Crops Prod..

[106] Moohan J., Stewart S.A., Espinosa E., Rosal A., Rodríguez A., Larrañeta E., Donnelly R.F., Domínguez-Robles J. (2020). Cellulose Nanofibers and Other Biopolymers for Biomedical Applications. A Review. Appl. Sci..

[107] Global Cellulose Nanofibers Market and Future. https://nanografi.com/blog/global-cellulose-nanofibers-market-and-future/?srsltid=AfmBOoqCRZo7mAHE-Mct_e_7LMfC9vZDldkqMge7AxsEMqqPagB-lZHB.

[108] Nanotechnology: Emerging Applications of Cellulose-Based Green Magnetic Nanocomposites | US Forest Service Research and Development. https://research.fs.usda.gov/treesearch/40282.

[109] Creating Eco-Friendly Materials by Combining Cellulose Nanofibers and Pineapple Leaf Fibers in Epoxy Composites. https://phys.org/news/2023-06-eco-friendly-materials-combining-cellulose-nanofibers.html#google_vignette.

[110] The Promise of Cellulose Nanofibers | Nippon.Com. https://www.nippon.com/en/behind/l00151/.

[111] Tibolla H., Pelissari F.M., Menegalli F.C. (2014). Cellulose Nanofibers Produced from Banana Peel by Chemical and Enzymatic Treatment. LWT—Food Sci. Technol..

[112] George J., Sabapathi S.N. (2015). Cellulose Nanocrystals: Synthesis, Functional Properties, and Applications. Nanotechnol. Sci. Appl..

[113] Juan L.-T., Lin S.-H., Wong C.-W., Jeng U.-S., Huang C.-F., Hsu S. (2022). Functionalized Cellulose Nanofibers as Crosslinkers to Produce Chitosan Self-Healing Hydrogel and Shape Memory Cryogel. ACS Appl. Mater. Interfaces.

[114] White M.S., Kaltenbrunner M., Głowacki E.D., Gutnichenko K., Kettlgruber G., Graz I., Aazou S., Ulbricht C., Egbe D.A.M., Miron M.C. (2013). Ultrathin, Highly Flexible and Stretchable PLEDs. Nat. Photonics.

[115] Huang Y., Zheng M., Lin Z., Zhao B., Zhang S., Yang J., Zhu C., Zhang H., Sun D., Shi Y. (2015). Flexible Cathodes and Multifunctional Interlayers Based on Carbonized Bacterial Cellulose for High-Performance Lithium–Sulfur Batteries. J. Mater. Chem. A.

[116] Moud A.A. (2022). Advanced Cellulose Nanocrystals (CNC) and Cellulose Nanofibrils (CNF) Aerogels: Bottom-up Assembly Perspective for Production of Adsorbents. Int. J. Biol. Macromol..

[117] Sun H., Que Z., Wei H., Zhou A., Peng X., Cui W., Wang X. (2022). Tunning Matrix Rheology and Mechanical Performance of Ultra-High Performance Concrete Using Cellulose Nanofibers. Nanotechnol. Rev..

[118] Li Y., Zhu H., Wang Y., Ray U., Zhu S., Dai J., Chen C., Fu K., Jang S.-H., Henderson D. (2017). Cellulose-Nanofiber-Enabled 3D Printing of a Carbon-Nanotube Microfiber Network. Small Methods.

[119] High-Performance Cellulose Nanofibril Composite Films: BioResources. https://bioresources.cnr.ncsu.edu/resources/high-performance-cellulose-nanofibril-composite-films/.

[120] Kalia S., Boufi S., Celli A., Kango S. (2014). Nanofibrillated Cellulose: Surface Modification and Potential Applications. Colloid Polym. Sci..

[121] Mohan D., Teong Z.K., Bakir A.N., Sajab M.S., Kaco H. (2020). Extending Cellulose-Based Polymers Application in Additive Manufacturing Technology: A Review of Recent Approaches. Polymers.

[122] Cellulose Nanofibers: SHIMADZU (Shimadzu Corporation). https://www.shimadzu.com/an/industries/engineering-materials/cellulose_nanofibers/index.html.

[123] Haq F., Kiran M., Khan I.A., Mehmood S., Aziz T., Haroon M. (2025). Exploring the Pathways to Sustainability: A Comprehensive Review of Biodegradable Plastics in the Circular Economy. Mater. Today Sustain..

[124] Arun R., Shruthy R., Preetha R., Sreejit V. (2022). Biodegradable Nano Composite Reinforced with Cellulose Nano Fiber from Coconut Industry Waste for Replacing Synthetic Plastic Food Packaging. Chemosphere.

[125] Nechyporchuk O., Belgacem M.N., Bras J. (2016). Production of Cellulose Nanofibrils: A Review of Recent Advances. Ind. Crops Prod..

[126] Foroughi F., Rezvani Ghomi E., Morshedi Dehaghi F., Borayek R., Ramakrishna S. (2021). A Review on the Life Cycle Assessment of Cellulose: From Properties to the Potential of Making It a Low Carbon Material. Materials.

[127] Ansari M.M., Heo Y., Do K., Ghosh M., Son Y.-O. (2024). Nanocellulose Derived from Agricultural Biowaste By-Products–Sustainable Synthesis, Biocompatibility, Biomedical Applications, and Future Perspectives: A Review. Carbohydr. Polym. Technol. Appl..

[128] Liu Y., Zhu W., Li Z., Xin R., He Y., Yang J., Li S., Chen M. (2024). Bamboo-Based Cellulose Nanofibers as Reinforcement for Polyurethane Imitation Wood. Ind. Crops Prod..

[129] Nanocellulose Market Size, Share, Value & Outlook by 2033. https://www.futuremarketinsights.com/reports/nanocellulose-market.

[130] Djafari Petroudy S.R., Chabot B., Loranger E., Naebe M., Shojaeiarani J., Gharehkhani S., Ahvazi B., Hu J., Thomas S. (2021). Recent Advances in Cellulose Nanofibers Preparation through Energy-Efficient Approaches: A Review. Energies.

[131] Signori-Iamin G., Santos A.F., Corazza M.L., Aguado R., Tarrés Q., Delgado-Aguilar M. (2022). Prediction of Cellulose Micro/Nanofiber Aspect Ratio and Yield of Nanofibrillation Using Machine Learning Techniques. Cellulose.

[132] Zeng J., Zeng Z., Cheng Z., Wang Y., Wang X., Wang B., Gao W. (2021). Cellulose Nanofibrils Manufactured by Various Methods with Application as Paper Strength Additives. Sci. Rep..

[133] Shaghaleh H., Xu X., Wang S. (2018). Current Progress in Production of Biopolymeric Materials Based on Cellulose, Cellulose Nanofibers, and Cellulose Derivatives. RSC Adv..

[134] Nanofibers Market Size, Share, Growth, Trends Report, 2030. https://www.grandviewresearch.com/industry-analysis/nanofibers-market.

[135] Sustainable Materials in Automotive Manufacturing: Trends and Innovations—Technology Innovators. https://www.technology-innovators.com/sustainable-materials-in-automotive-manufacturing-trends-and-innovations/.

[136] James A., Rahman M.R., Mohamad Said K.A., Kanakaraju D., Sueraya A.Z., Kuok K.K., Bin Bakri M.K., Rahman M.M. (2024). A Review of Nanocellulose Modification and Compatibility Barrier for Various Applications. J. Thermoplast. Compos. Mater..

